# Doxorubicin-Loaded Poly(Substituted Glycolide)-Based Nanoparticles for Long-Term Storage

**DOI:** 10.1021/acsomega.6c01126

**Published:** 2026-06-06

**Authors:** Tuğba Koldankaya, Mehmet Onur Arıcan, Olcay Mert, Serap Mert

**Affiliations:** † Department of Polymer Science and Technology, 52980 Kocaeli University, 41001 Kocaeli, Turkey; ‡ Department of Chemistry, Faculty of Arts and Sciences, Kocaeli University, 41001 Kocaeli, Turkey; § Center for Stem Cell and Gene Therapies Research and Practice, Kocaeli University, 41001 Kocaeli, Turkey

## Abstract

Preventing agglomeration is crucial for polymeric nanoparticles (PNP) for drug-delivery applications. Suitable conditions for maintaining the size of doxorubicin (DOX)-loaded poly­(diisobutyl glycolide) (PDIBG) and poly­(diisopropyl glycolide) (PDIPG) nanoparticles (NP) during long-term storage were investigated in this study. After the synthesis of PDIBG and PDIPG homopolymers, DOX-loaded NPs were produced from these homopolymers by using a single-emulsion solvent evaporation method. The optimal formulations of DOX-loaded PDIBG and PDIPG-NPs were obtained with particle sizes of 253 ± 7 nm, PDIs of 0.06 ± 0.02, and an EE value of 58.3%, and particle sizes of 253 ± 7 nm, PDIs of 0.08 ± 0.04, and an EE value of 73.9%, respectively. To extend their shelf life, the developed NPs at three different concentrations (50, 70, and 90 mg/mL) were subjected to a comprehensive lyophilization process at two different freezing temperatures (−20 and −50 °C). As a result of the systematic lyophilization of sugar-containing and sugar-free formulations of DOX-loaded PDIBG and PDIPG-NPs under specific conditions, it was found that they could be stored at 4 and −20 °C for 2 months while maintaining their particle sizes and PDI values in the presence of glucose and sucrose (at 5 and 10% concentrations).

## Graphical abstract

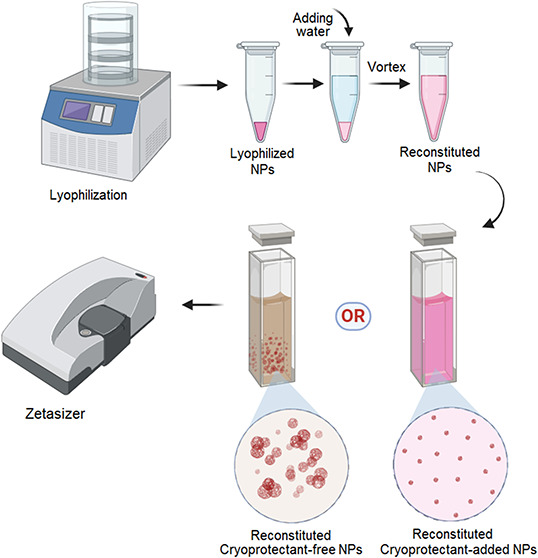

## 1 Introduction

Doxorubicin (DOX), an anticancer drug from the anthracycline family, is used in the treatment of various types of cancer, particularly breast cancer, and offers great potential in the early stages of disease.^1,2^ However, the traditional nonspecific use of DOX causes undesirable side effects in patients (such as nausea, vomiting, fatigue, and cardiotoxicity), and the clinical use of DOX is limited due to dose-dependent cardiotoxicity, preventing the use of high-concentration dosages.^1,3–5^ Nanobased carrier systems developed to eliminate these disadvantages can increase the therapeutic efficacy of DOX, prevent its degradation during circulation in the body, minimize side effects, and enable controlled drug release by increasing local drug concentration.^3,6,7^ With the advances in nanotechnology, studies on drug-delivery systems for cancer treatment have focused on the production and improvement of various formulations.^8^ Several studies have been conducted on the production of various nanocarrier systems containing DOX, including inorganic NPs such as gold^9^ and iron oxide,^10,11^ and organic NPs such as polymeric NPs,^6,12,13^ liposomes,^14,15^ micelles,^16,17^ and micro/nanoemulsions.^2,18–20^

Polymeric nanoparticles (PNPs) offer significant advantages over liposomes, such as enhancing the stability of drugs and proteins and enabling controlled release. PNPs, which possess the essential properties of biodegradability and biocompatibility for pharmaceutical products, are widely preferred in drug delivery because they break down into harmless monomeric components when degraded in the body. PNPs enable the production of formulations with controllable size, shape, and surface charge, which can be chemically and biologically functionalized to facilitate targeted drug delivery. Furthermore, because of their ability to respond to stimuli associated with specific biological environments, effective drug release can be achieved.^21,22^

Polylactide (PLA), poly­(lactide-*co*-glycolide) (PLGA), and poly­(ε-caprolactone) (PCL) are members of the poly­(substituted glycolides) family and are widely used in FDA-approved formulations due to their excellent biocompatibility, biodegradability, low toxicity, and ability to provide effective and controlled drug release.^23,24^ These polymers are frequently used in drug-delivery systems, and among them, PLGA is particularly popular due to its use in many studies.^13,25,26^ Numerous researchers have favored PLGA for the encapsulation of DOX^27^ and have employed different techniques, such as single-emulsion solvent evaporation,^28–31^ double-emulsion solvent evaporation,^13,32–34^ and the electrospray technique.^3^ In addition to these widely studied conventional polymers, recent advances have highlighted the potential of other novel biodegradable aliphatic polyesters. For instance, recent pioneering studies have demonstrated the high effectiveness of mPEG-functionalized polyadipate triblock copolymers as robust nanocarriers for the controlled delivery of DOX in breast cancer models.^35,36^ These systems have shown that precisely tuning the polymer’s aliphatic chain structure can significantly optimize drug encapsulation efficiency (EE), sustained release profiles, and cellular uptake. Inspired by the success of such structurally tunable, biodegradable polyester-based nanocarriers, poly­(substituted glycolides) (PSGs) emerge as highly promising candidates. PSGs offer many advantages, due to the structural similarity of^37^ polyglycolic acid (PGA) and PLA, which are frequently used in biological applications and known for their biocompatibility and biodegradability.^38–40^ These include micelle formation and high drug-loading capacities,^39,41,42^ enabled by the designed substituent groups on the polymer chain. As a result, the synthesis and polymerization of substituted glycolides have become increasingly popular and are rapidly being developed to provide new perspectives for various biological applications.^38,40–45^ Given these advantages, it represents a viable alternative to conventional polymers, such as PLGA, PGA, and PLA, which are widely used in medical applications, particularly in drug-delivery systems.

Lyophilization, a freeze-drying process, is required to ensure the stability and extended shelf life of pharmaceutical NPs during storage and transportation. This process involves removing water from the frozen sample under a low vacuum.^46,47^ The lyophilization process prevents NP aggregation and degradation that may occur when they remain in an aqueous environment while also offering a practical and safe application through the reconstitution of the resulting dry product.^46,48^ However, depending on their surface properties, shapes, and sizes, NPs are subjected to various stresses during the freezing process.^47^ One of these is stress caused by ice crystals, which cause NP particles to cluster together at the ice boundary.^49^ To overcome these issues and ensure successful freeze-drying by forming a protective barrier around the sample, sugars such as mannitol, sucrose, and glucose are commonly used as cryoprotectants.^12,50^ Consequently, the selection of an appropriate cryoprotectant and its optimal concentration is critical for effective protection.^12,46^

In various studies, the synthesis of PEG/mPEG-based copolymers has been synthesized via ring-opening polymerization of diisobutyl and/or diisopropyl-substituted glycolide monomers^41,42,51^ and the corresponding homopolymers,^52^ as well as symmetric star-shaped homopolymers,^44^ were also prepared. A common feature of these studies is their contribution to the literature by elucidating the structure–property relationships in polymers derived from substituted glycolides; in these studies, emphasis is placed on the modulation of surface properties and thermosensitive behavior in selected systems through the precise control of polymer architecture and composition. In this study, poly­(diisobutyl glycolide) (PDIBG) and poly­(diisopropyl glycolide) (PDIPG) homopolymers belonging to the PSG family were synthesized using a previously established synthetic approach (Figure 2) and subsequently employed for the development of DOX-loaded PDIPG and PDIBG nanoparticles (NPs) (Figure 1). To the best of our knowledge, this study constitutes the first report describing the freeze-drying processes of PDIPG and PDIBG homopolymers for use in drug-delivery systems, with particular emphasis on a comprehensive and comparative evaluation of the lyophilization conditions and the subsequent storage stability of the developed NP formulations. By adjusting polymer concentrations, the nanoformulations were optimized to achieve precise control over particle size and polydispersity index (PDI). A primary objective of this work was to establish robust storage protocols, including suitable cryoprotectants, storage temperature, and duration, to maintain the physical stability of DOX-PSG-NPs without aggregation. To this end, a systematic lyophilization study was conducted to evaluate the cryoprotective efficacy of mannitol, glucose, and sucrose at varying concentrations (1%, 5%, and 10%). In addition, the long-term post-storage stability of reconstituted DOX-PSG-NPs was assessed for up to two months at 4 °C and −20 °C to determine optimal storage conditions. Finally, *in vitro* drug release studies were performed to elucidate the influence of alkyl side-chain architecture in the PSG backbone on the DOX release profile.

**Figure 1:**
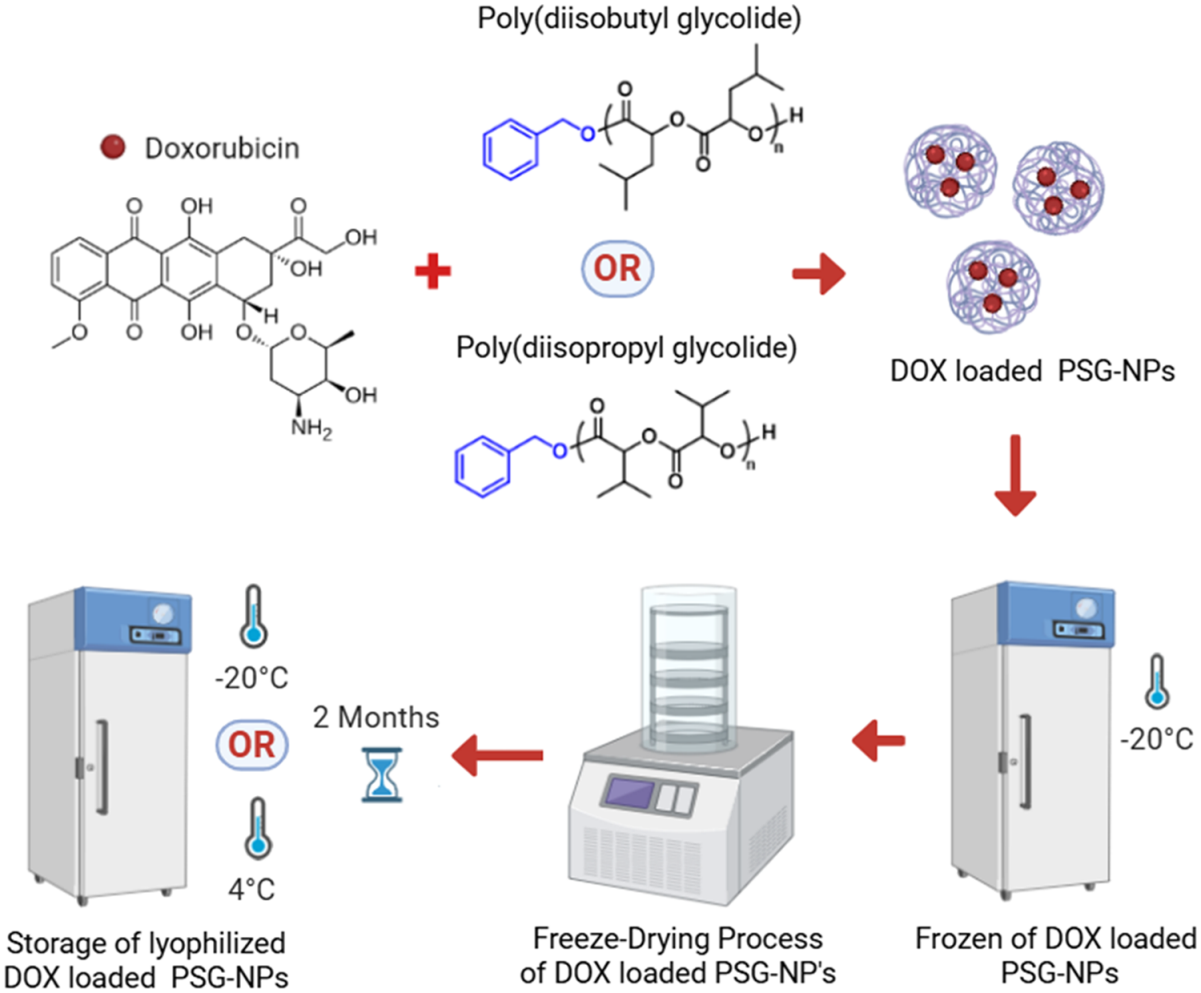
Production of nanoparticles based on poly­(substituted glycolide) containing DOX.

## 2 Materials and Methods

### 2.1 Materials

l-Leucine (l-2-amino-4-methylpentanoic acid) (TCI, 99%) and l-valine (l-2-amino-3-methylbutanoic acid) (Sigma-Aldrich, 98%) amino acids were used for the synthesis of l-2-hydroxy-4-methylpentanoic acid (l-isobutyl hydroxy acid) and l-2-hydroxy-3-methylbutanoic acid (l-isopropyl hydroxy acid), respectively. Sodium nitrite (NaNO_2_) (BDH Chemicals) and sulfuric acid (H_2_SO_4_) (Sigma-Aldrich, 95–97%) were also used for α-hydroxy acid synthesis. l-3,6-diisopropyl-1,4-dioxane-2,5-dione (l-diisopropyl glycolide, LDIPG) and l-3,6-diisobutyl-1,4-dioxane-2,5-dione (l-diisobutyl glycolide, LDIBG) monomers were synthesized using p-toluenesulfonic acid monohydrate (PTSA·H_2_O) (Sigma-Aldrich, 98.5%) as a catalyst in toluene (Sigma-Aldrich, 99.7%). In the synthesis of PDIBG and PDIPG homopolymers, tin­(II) 2-ethylhexanoate (tin­(II) octoate, Sn­(Oct)_2_, Sigma-Aldrich, 95%) and benzyl alcohol (Sigma-Aldrich, 99.8%) were used as catalyst and initiator, respectively. For the preparation of NPs, poly­(vinyl alcohol) (PVA, Mowiol, MW ∼ 31 kDa) and triethylamine (Et_3_N) (Sigma-Aldrich, 99%) were used. Mannitol (BDH Chemicals), d-glucose (Galenik Pharmaceuticals), and sucrose (Merck) were used as cryoprotectants in the lyophilization studies of the NPs. Dichloromethane (99.5%), dimethyl sulfoxide (99.9%), and methanol (99.8%) were obtained from Sigma-Aldrich and were used during the preparation of PSG-NPs and UV analyses.

### 2.2 Characterization

The α-hydroxy acids, monomers, and homopolymers were structurally characterized using Attenuated Total Reflectance-Fourier Transform Infrared (ATR-FTIR, ATR Bruker-Tensor 27) and Nuclear Magnetic Resonance (NMR, Bruker Avance III 400 MHz) spectrometers. The molecular weight and polydispersity of PDIPG and PDIBG homopolymers were characterized by Gel Permeation Chromatography (GPC, Malvern/Viscotek RI max system). Specific optical rotation measurements of substituted glycolide monomers and polymers were carried out (*c* = 0.25 g per 100 mL in chloroform at 20 °C) using an MCP-150 polarimeter (Anton Paar). HPLC analyses of substituted glycolide monomers were performed using an Agilent 1260 Infinity system equipped with a UV detector (200 nm) and a reverse-phase ZORBAX SB-C18 column (4.6 × 150 mm^2^, 3.5 μm). The chromatographic conditions were carried out using a methanol mobile phase at a flow rate of 1.0 mL/min, a column temperature of 40 °C, and an injection volume of 20 μL.

The particle size and PDI value of the prepared PSG-NPs were determined using dynamic light scattering (DLS, Zetasizer Nano ZS 90). Scanning electron microscopy (SEM) was used for morphological analysis of the lyophilized NPs. Three replicate measurements were performed for each sample in particle size analyses.

Differential scanning calorimetry (DSC, Mettler Toledo DSC1 Star System) was used for the thermal characterization of PSG homopolymers, PSG-NPs, and DOX-loaded PSG-NPs. Thermogravimetric analysis (TGA, 1 Star system) was performed to determine the mass loss occurring with temperature in PSG homopolymers.

### 2.3 Synthesis of l-2-Hydroxy-4-Methylpentanoic Acid (Isobutyl Hydroxy Acid, IBHA) and l-2-Hydroxy-3-Methylbutanoic Acid (Isopropyl Hydroxy Acid, IPHA)

l-2-Hydroxy-4-methylpentanoic acid and l-2-hydroxy-3-methylbutanoic acid were synthesized using the literature-known protocol previously performed by our group.^37,41,42,44,51,52^ l-Leucine (l-2-amino-4-methylpentanoic acid; 13.25 g, 0.1 mol) amino acid was combined with 80 mL of 2.5 M H_2_SO_4_ (0.2 mol) solution, and the mixture was cooled to 0 °C using an ice bath. Then, 80 mL of sodium nitrite solution was added drop by drop using a dropping funnel under a fume hood. After this process was completed, the reaction mixture was stirred at the same temperature for 3 h and then at room temperature overnight. At the end of the mixing time, the solution was saturated with NaCl and extracted with 80 mL of diethyl ether. The extraction process was repeated three times, and the diethyl ether organic phase was dried with sodium sulfate. The organic solvent was removed, and the resulting yellow viscous product was precipitated and crystallized with diethyl ether and hexane, yielding white crystals of l-2-hydroxy-4-methylpentanoic acid (IBHA) with approximately 52% yield. The same protocol was applied to the l-valine (l-2-amino-3-methylbutanoic acid; 11.95 g, 0.1 mol) amino acid, yielding l-2-hydroxy-3-methylbutanoic acid (IPHA) with an approximate 48% yield. IBHA: ^1^H NMR (400 MHz, CDCl_3_) δ: 0.95 (d, *J* = 8.4 Hz, 6H, 2 × C*H*_3_), 1.61 (dist. t, *J* = 7.2 Hz, 2H, C*H*_2_), 1.88 (m, 1H, C*H*), 4.28 (dd, *J* = 6.0, 7.1 Hz, 1H, C*H*), 6.36–8.19 (br, 2H, OH, COO*H*); ^13^C NMR (100 MHz, CDCl_3_) δ: 21.39, 23.15, 24.44, 43.10, 68.96, 179.89; and ATR-FTIR (ν_max_/cm^–1^): 1701 (C=O), 2874, 2905, 2934, 2957 (CH), 3420 (OH). IPHA: ^1^H NMR (400 MHz, CDCl_3_) δ: 0.89 (d, *J* = 6.8 Hz, 3H, C*H*_3_), 1.02 (d, *J* = 6.9 Hz, 3H, C*H*_3_), 2.12 (m, 1H, C*H*), 4.14 (d, *J* = 3.5 Hz, 1H, C*H*), 6.01–9.00 (br, 2H, O*H*, COO*H*); ^13^C NMR (100 MHz, CDCl_3_) δ: 15.89, 18.71, 31.95, 74.89, 179.06; ATR-FTIR (ν_max_/cm^–1^): 1701 (C=O), 2972, 2935, 2934, 2880 (CH), 3413 (OH).

### 2.4 Synthesis of l-3,6-Diisobutyl-1,4-Dioxane-2,5-Dione (l-Diisobutyl Glycolide, LDIBG) and l-3,6-Diisopropyl-1,4-Dioxane-2,5-Dione (l-Diisopropyl Glycolide, LDIPG) Monomers

Glycolide monomers were synthesized by the literature-known protocol previously performed by our group.^37,41,44,51,52^ A reflux system was set up using a Dean–Stark apparatus for monomer synthesis; thus, the water released during the formation of the monomer ring was removed. The synthesis of LDIBG monomer was carried out by mixing IBHA (10 g, 75.7 mmol) and PTSA. H_2_O (200 mg, 1.05 mmol) in toluene (200 mL) at 140 °C for 12 h. After removing toluene, the resulting monomer residue was washed three times with diethyl ether and then dried, yielding LDIBG with a 39% yield.^41,51,52^ The same protocol was applied with IPHA (10.5 g, 88.2 mmol), and after 42 h of stirring, LDIPG monomer was obtained with 36% yield. LDIBG: ^1^H NMR (400 MHz, CDCl_3_) δ: 0.96 (d, *J* = 6.0 Hz, 6H, 2 × C*H*_3_), 0.99 (d, *J* = 6.0 Hz, 6H, 2 × C*H*_3_), 1.92 (m, 6H, 2 × C*H*, 2 × C*H*_2_), 4.92 (dd, *J* = 3.7, 8.8 Hz, 2H, 2 × C*H*); ^13^C NMR (100 MHz, CDCl_3_) δ: 21.29, 23.02, 23.85, 38.82, 74.20, 167.37; ATR-FTIR (ν_max_/cm^–1^): 1755 (C=O), 2868, 2928, 2957 (CH). [α]_D_: −238.1.^41,53^ LDIPG: ^1^H NMR (400 MHz, CDCl_3_) δ: 1.03 (d, *J* = 6.8 Hz, 6H, 2 × C*H*_3_), 1.13 (d, *J* = 7.0 Hz, 6H, 2 × C*H*_3_), 2.48 (m, 2H, 2 × C*H*), 4.71 (d, *J* = 3.1 Hz, 2H, 2 × C*H*); ^13^C NMR (100 MHz, CDCl_3_) δ: 15.84, 18.52, 29.41, 79.59, 166.43; ATR-FTIR (ν_max_/cm^–1^): 1748 (C=O), 2969, 2939, 2878 (CH). [α]_D_: −268.1.^53,54^

### 2.5 Synthesis of Poly­(Diisobutyl Glycolide) (PDIBG) and Poly­(Diisopropyl Glycolide) (PDIPG) Homopolymers

The synthesis of substituted glycolide homopolymers was carried out in bulk at 175 °C under an argon atmosphere for 6 h. PDIBG homopolymer was synthesized by ring-opening polymerization of LDIBG as the monomer, tin­(II) 2-ethylhexanoate as the catalyst, and benzyl alcohol as the initiator in a 50:1:1 (monomer: initiator: catalyst) ratio.

At the end of the mixing period, the resulting polymers were dissolved in a small amount of dichloromethane, precipitated by adding methanol, kept overnight at −20 °C, separated from excess solvent using a centrifuge at −20 °C, and completely dried using an evaporator.^41,51,52^ The same protocol was applied for the synthesis of PDIPG homopolymer, and polymerization of LDIPG was carried out at 185 °C for 8 h. PDIBG: ^1^H NMR (400 MHz, CDCl_3_) δ: 0.92 (d, *J* = 5.9 Hz, 6H, 2 × C*H*_3_), 0.96 (d, *J* = 5.7 Hz, 6H, 2 × C*H*_3_), 1.77 (m, 6H, 2 × C*H*, 2 × C*H*_2_), 5.08 (dd, *J* = 3.4 Hz, 8.8 Hz, 2H, 2 × C*H*), 7.30–7.41 (m, 5H, 5 x *CH*); ^13^C NMR (100 MHz, CDCl_3_) δ: 21.44, 22.95, 24.53, 39.34, 71.37, 169.71; ATR-FTIR (ν_max_/cm^–1^): 1753 (C=O), 2873, 2958 (CH). [α]_D_: −81.2.^53^ PDIPG: ^1^H NMR (400 MHz, CDCl_3_) δ: 1.04 (app. t (overlapped d), *J* = 7.0 Hz, 12H, 4 × C*H*_3_), 2.36 (m, 2H, 2 × CH), 5.00 (d, *J* = 3.6 Hz, 2H, 2 × CH), 7.36 (m, 5H, 5 × *CH*); ^13^C NMR (100 MHz, CDCl_3_) δ: 16.99, 18.69, 30.35, 76.98, 168.83; ATR-FTIR (ν_max_/cm^–1^): 1749 (C=O), 2936, 2973, 2880 (CH). [α]_D_: −69.6.^53^

### 2.6 Preparation of DOX-Loaded PSG Nanoparticles

DOX-loaded PDIPG-NPs and PDIBG-NPs were prepared using the single-emulsion solvent evaporation method, with minor modifications to a previously reported protocol for DOX-PLGA-NPs.^12^ When preparing DOX-PSG-NPs, different polymer amounts of 50, 70, and 100 mg were used to determine the optimal polymer-to-drug ratio. The polymer was dissolved in 2 mL of dichloromethane and mixed with 0.2 mL of DOX solution (3 mg DOX/20 μL TEA/1 mL methanol). The resulting organic phase was emulsified with 5 mL of PVA (1.25%) solution by sonication at 65% amplitude for 4 min in an ice bath. The resulting oil/water emulsion was transferred into 5 mL of PVA (1.25%) solution under magnetic stirring at 1000 rpm. The mixture was stirred overnight to allow solvent evaporation, followed by centrifugation at 15,000 rpm for 15 min at 4 °C. The obtained NPs were washed twice with distilled water under the same centrifugation conditions.^12,34,55^

### 2.7 Determination of Encapsulation Efficiency and Drug-Loading Content

A UV–vis spectrophotometer was used to determine the amount of DOX encapsulated in the PSG-NPs.^12,56,57^ Calibration curves were prepared by using five different DOX concentrations (125, 62.5, 31.25, 15.63, and 7.81 μg/mL) in dimethyl sulfoxide (DMSO) and dichloromethane (DCM), respectively, and measuring the absorbance at 480 nm. DOX-loaded PDIPG-NPs were dissolved in DMSO, while DOX-loaded PDIBG-NPs were dissolved in DCM. The amount of encapsulated DOX in the NPs was calculated using the corresponding calibration curve obtained in the appropriate solvent. EE and drug-loading (DL) percentage of DOX-PSG-NPs were calculated using eqs 1 ^58^ and 2,^59^ respectively.

$$\mathrm{EE}\%=\frac{\mathrm{Amount of DOX in NP}}{\mathrm{Total amount of DOX}}\times 100$$ 1

2 $$\mathrm{DL}\%=\frac{\mathrm{Amount of DOX in NP}}{\mathrm{Amount of NP}}\times 100$$

### 2.8 Release of DOX from PSG-NPs

The drug release profiles of DOX-PDIPG-NP and DOX-PDIBG-NP were comparatively evaluated. Lyophilized NPs were suspended in 1 mL of PBS (pH 7.4) and incubated at 37 °C under continuous stirring at 100 rpm for up to 360 h. At predetermined time points (0, 1, 24, 48, ..., 360 h), NP suspensions were centrifuged at 10,000 rpm for 12 min. The pellet obtained after centrifugation was vortexed until it was completely dispersed. The entire upper phase (1 mL) was collected and replaced with an equal volume of fresh PBS to maintain sink conditions. After the lyophilization of the collected supernatants, the residues were dissolved in DMSO, and the amount of released DOX was quantified by UV–visible spectrophotometry at 480 nm. Drug release experiments were performed in duplicate (*n* = 2) for DOX release from PDIBG- or PDIPG-NPs, and each sample was analyzed in triplicate. Mean absorbance values at 480 nm and their corresponding standard deviations (SD values) were calculated. The dilution factor of the samples (final volume of 3 mL in the UV cuvette) was taken into account when calculating the cumulative drug release.

### 2.9 Freeze-Drying Process

During the lyophilization of DOX-PSG-NPs, water was removed from the frozen NPs using a lyophilizer (Labconco FreeZone Plus Cascade Freeze-Dry Systems 2.5 L) at −80 °C under a vacuum of 0.010 mbar. The lyophilized NPs were reconstituted in water to their original volumes, and particle size analyses were performed by DLS. Error bars in the DLS size distribution graphs represent the standard deviation of the measurements (*n* = 2 independent samples, with 3 replicates each).

#### 2.9.1 Use of Different Freezing Temperatures

DOX-PSG-NPs were mixed with equal volumes of sugar concentrations to reach final 1%, 5%, or 10% mannitol, glucose, and sucrose solutions and then frozen at −20 °C for one night or −50 °C for 3 h, before lyophilization. The two freezing temperatures were selected to investigate their effect on particle stability, taking into account the *T*_g_^’^ values of the sugars used during lyophilization.^12^

#### 2.9.2 Use of Different NP Concentrations

DOX-PSG-NPs were prepared with final 5% or 10% glucose or sucrose solutions to achieve final NP concentrations of 50, 70, and 90 mg/mL and frozen at −20 °C before lyophilization. The effects of different DOX-PSG-NP concentrations on particle size and polydispersity index (PDI) after lyophilization were subsequently evaluated. The lyophilized NPs were redispersed in water and characterized by DLS.

### 2.10 Physical Stability and Redispersibility of DOX-PSG-NPs After Storage

DOX-PSG-NPs were lyophilized at a final concentration of 50 mg/mL in the presence of 5% or 10% glucose or sucrose and subsequently stored at two different temperatures, 4 and −20 °C, for 2 months. Storage tests conducted with two replicate samples (*n* = 2) were performed at the end of the storage period, where lyophilized NPs were rehydrated with water, and particle size analysis was performed in triplicate. The error bars in the DLS size distribution plots for the stability tests represent the SD values.

### 2.11 *In Vitro* Degradation of PSG-NPs

The *in vitro* degradation of PSG-NPs was carried out based on studies reported in the literature^37,41,60^ by incubating 8 mg of PDIBG and PDIPG-NPs in 1 mL of PBS (pH 7.4) at 37 °C and a stirring speed of 200 rpm. Degradation samples collected at specific time intervals (0, 4, 12, 20, 28, and 35 days) were lyophilized after being centrifuged at 15,000 rpm for 15 min. The degradation experiments, carried out in triplicate, were analyzed by GPC.

## 3 Results and Discussion

### 3.1 Synthesis and Characterization of Substituted Glycolide Monomers

The syntheses of the LDIBG and LDIPG monomers were carried out in two main stages for each compound. In the first stage, l-2-hydroxy-4-methylpentanoic acid (IBHA) was obtained from l-leucine (l-2-amino-4-methylpentanoic acid) via diazotization (Figure 2). In the second stage, IBHA was converted to the LDIBG monomer through refluxing in toluene in the presence of PTSA.H_2_O.^37,41,42,44,52^ Similarly, l-2-hydroxy-3-methylbutanoic acid (IPHA) was synthesized from l-valine (l-2-amino-3-methylbutanoic acid) and subsequently refluxed under identical conditions to yield the LDIPG monomer.^37,41,42,51^ Structural characterization of synthesized hydroxy acids and monomers was performed using ^1^H NMR, ^13^C NMR, and ATR-FTIR spectroscopy techniques and is shown in Figure S1.

**Figure 2:**
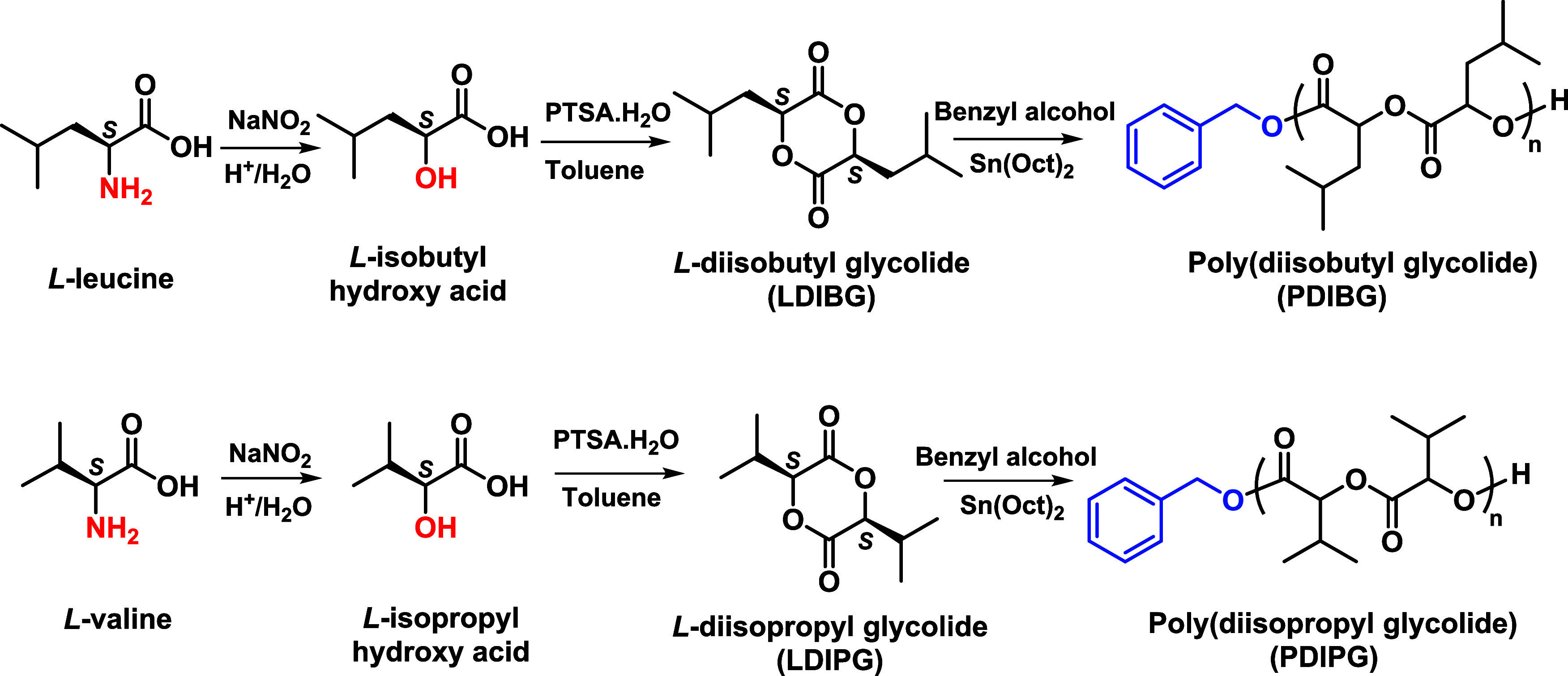
Syntheses of hydroxy acids, monomers, and PSG homopolymers.

The ^1^H NMR spectrum of IBHA (Figure S1A) shows characteristic signals consistent with the expected structure. A doublet peak at 0.95 ppm corresponds to the methyl protons labeled (a) and (b), while a distorted triplet at 1.61 ppm is assigned to the methylene proton labeled (c). The methine proton (d) appears as a multiplet at 1.88 ppm, and the α-methine proton (e) is observed as a doublet of doublets at 4.28 ppm. Broad signals between 6.36 and 8.19 ppm correspond to the hydroxyl protons (f and g). In the ^13^C NMR spectrum of IBHA (Figure S1B), the methyl carbons appear at 21.39 ppm (a) and 23.15 ppm (b), the methine carbon (d) at 24.44 ppm, the methylene carbon (c) at 43.10 ppm, the α-methine carbon (e) at 68.96 ppm, and the carbonyl carbon of the carboxylic acid group (h) at 179.89 ppm. These spectral data are in good agreement with previously reported results.^37,41,42,44,52^ The ATR-FTIR spectrum of IBHA (Figure S1C) further confirms successful synthesis. The broad peak at 3420 cm^–1^ corresponds to the hydroxyl (OH) stretching vibration, which differs from that of the l-leucine starting material due to the formation of a new hydroxyl environment. C–H stretching vibrations are observed at 2957, 2934, 2905, and 2874 cm^–1^, while a sharp peak at 1701 cm^–1^ is attributed to the carbonyl (C=O) stretching of the carboxylic acid group. Collectively, the NMR and ATR-FTIR data confirm the successful conversion of l-leucine to IBHA.

The ^1^H NMR spectrum of the LDIBG monomer (Figure S1A) exhibits two doublets at 0.96 and 0.99 ppm, corresponding to the methyl protons (a and b) of the substituted isobutyl group on the glycolide ring. The methine and methylene protons (d and c) overlap around 1.92 ppm, while the α-methine proton (e) appears as a doublet at 4.92 ppm. Notably, the C*H* proton of the IBHA starting material, originally observed at 4.28 ppm, shifts to 4.92 ppm in the monomer due to chemical modification during ring closure. Additionally, the disappearance of the hydroxyl proton signals (6.36–8.19 ppm) present in IBHA confirms successful esterification and monomer formation. In the ^13^C NMR spectrum (Figure S1B), the methyl carbons a and b are observed at 21.29 and 23.02 ppm, respectively, while the methine carbon d appears at 23.85 ppm and the methylene carbon c at 38.82 ppm. The α-methine carbon e resonates at 74.20 ppm, and the carbonyl carbon f of the ester is observed at 167.37 ppm. Compared to the IBHA starting material, which exhibited peaks at 68.96 and 179.89 ppm for the α-methine and carboxyl carbons, respectively, these shifts reflect the formation of the cyclic ester through ring closure. The ^1^H and ^13^C NMR data are consistent with previously reported literature.^37,41,42,44,52^ The ATR-FTIR spectrum of LDIBG (Figure S1C) further confirms monomer formation. A sharp peak at 1755 cm^–1^ corresponds to the C=O stretching vibration of the ester group. C–H stretching vibrations are observed at 2969, 2939, and 2878 cm^–1^. The broad absorption band observed at 3420 cm^–1^, attributed to the hydroxyl group present in the IBHA starting material, is absent in the LDIBG spectrum, indicating successful ring closure. Collectively, the NMR and ATR-FTIR data confirm the successful synthesis of the LDIBG monomer via cyclization of IBHA.

The ^1^H NMR spectrum of IPHA (Figure S1D) displays characteristic signals consistent with its expected structure. A doublet at 0.89 ppm corresponds to the methyl protons labeled a, while another doublet at 1.02 ppm is assigned to the methyl protons labeled b. The methine proton (c) appears as a multiplet at 2.12 ppm, and the α-methine proton (d) is observed as a doublet at 4.14 ppm. Broad signals between 6.01 and 9.00 ppm correspond to the hydroxyl protons (e and f). In the ^13^C NMR spectrum of IPHA (Figure S1E), the methyl carbons a and b resonate at 15.89 and 18.71 ppm, respectively, the methine carbon c at 31.95 ppm, the α-methine carbon d at 74.89 ppm, and the carbonyl carbon g at 179.06 ppm. These spectral data are in agreement with previously reported values.^37,44,51^ The ATR-FTIR spectrum of IPHA (Figure S1F) further confirms the structure. A sharp peak at 1701 cm^–1^ corresponds to the carbonyl (C=O) stretching vibration of the carboxylic acid group. C–H stretching vibrations are observed at 2972, 2934, and 2880 cm^–1^. A broad peak at 3413 cm^–1^, corresponding to the hydroxyl (OH) group, indicates a different hydroxyl environment compared with the l-valine starting material. These spectroscopic data collectively confirm the successful synthesis of IPHA, consistent with literature reports.^37,51^

The ^1^H NMR spectrum of the LDIPG monomer (Figure S1D) displays characteristic signals confirming successful monomer formation. A doublet at 1.03 ppm corresponds to the methyl protons labeled a, while another doublet at 1.13 ppm is assigned to the methyl protons labeled b. The methine protons (c) appear as a multiplet at 2.48 ppm, and the α-methine proton (d) is observed as a doublet at 4.71 ppm. Notably, the CH proton of the IPHA starting material, originally at 4.14 ppm, shifts to 4.71 ppm upon monomer synthesis, consistent with chemical modification during ring closure. In the ^13^C NMR spectrum (Figure S1E), the methyl carbons a and b resonate at 15.84 and 18.52 ppm, respectively, the methine carbon c at 29.41 ppm, the α-methine carbon d at 79.59 ppm, and the carbonyl carbon e of the ester appears at 166.43 ppm. Compared with the IPHA starting material, which exhibited peaks at 74.89 and 179.06 ppm for the α-methine and carbonyl carbons, respectively, these shifts reflect the formation of the cyclic ester upon ring closure. The ^1^H and ^13^C NMR data are consistent with previous reports.^37,44,51^ The ATR-FTIR spectrum of LDIPG (Figure S1F) further supports monomer formation. A sharp peak at 1748 cm^–1^ corresponds to the carbonyl (C=O) stretching of the ester group. C–H stretching vibrations are observed at 2969, 2939, and 2878 cm^–1^. In comparison, the IPHA starting material showed carbonyl stretching at 1702 cm^–1^, which shifts to 1748 cm^–1^ due to ester formation. Additionally, the disappearance of the hydroxyl (OH) stretching peak at 3413 cm^–1^ confirms successful ring closure and LDIPG monomer synthesis.^37,51^

To confirm the retention of the chiral carbon (*S* configuration) atoms, stereochemical analysis was performed using polarimetry. The specific optical rotation ([α]_D_) values of the synthesized monomers were recorded as −238.1° for LDIBG and −268.1° for LDIPG, confirming their high optical purity and consistency with the literature.^41,53,54^ The purity of the synthesized LDIBG and LDIPG monomers was further validated using high-performance liquid chromatography (HPLC), which confirmed that the highly pure monomers were successfully isolated (Figure S20).

### 3.2 Synthesis and Characterization of Poly­(Substituted Glycolide) Homopolymers

The homopolymers PDIBG and PDIPG were synthesized via ring-opening polymerization of LDIBG and LDIPG monomers, respectively, at 175–185 °C under solvent-free conditions, using benzyl alcohol as the initiator and Sn­(Oct)_2_ as the catalyst (Figure 2). Structural characterization of the resulting homopolymers was performed using ^1^H NMR, ^13^C NMR, and ATR-FTIR spectroscopy. Molecular weight and polydispersity index (PDI = *M*_w_/*M*_n_) were determined by gel permeation chromatography (GPC), with the results summarized in Table 1. Thermal properties of the polymers were assessed using differential scanning calorimetry (DSC) and thermogravimetric analysis (TGA).

**Table 1:** Properties of the Synthesised Poly­(Substituted Glycolide) Homopolymers

polymer type	*M*_n_^GPC^ (g/mol)	*M*_n_^NMR^ (g/mol)	*M*_n_^theoretical^ (g/mol)	*M*_w_/*M*_n_
PDIBG	12,000	12,350	11,410	1.45
PDIPG	8350	10,400	10,000	1.45

The ^1^H NMR spectrum of the PDIBG homopolymer (Figure S1A) exhibits characteristic signals confirming successful ring-opening polymerization. Doublets at 0.92 and 0.96 ppm correspond to the methyl protons (a and b) of the isobutyl group, while the methine (d) and methylene (c) protons of the isobutyl group overlap around 1.77 ppm. The α-methine protons of the PDIBG main chain (e) appear as a peak at 5.08 ppm, showing a downfield shift compared to the corresponding peak in the LDIBG monomer (from 4.92 to 5.08 ppm), consistent with polymer formation. A multiplet between 7.30 and 7.41 ppm (i) corresponds to the aromatic protons of the benzyl alcohol initiator. In the ^13^C NMR spectrum (Figure S1B), methyl carbons a and b resonate at 21.44 and 22.95 ppm, respectively, the methine carbon d at 24.53 ppm, the methylene carbon c at 39.34 ppm, the α-methine carbon e at 71.37 ppm, and the carbonyl carbon h at 169.71 ppm. Compared to the LDIBG monomer, in which the α-methine and carbonyl carbons appeared at 74.20 and 167.37 ppm, respectively, these shifts provide further evidence of successful ring-opening polymerization. The ATR-FTIR spectrum of PDIBG (Figure S1C) supports these findings. C–H stretching vibrations are observed at 2958 and 2873 cm^–1^, while the sharp peak at 1753 cm^–1^ corresponds to the carbonyl (C=O) stretching of the ester group. The slight shift in the carbonyl stretching frequency from 1755 cm^–1^ in the monomer to 1753 cm^–1^ in the polymer provides additional confirmation of successful polymerization. Collectively, the NMR and ATR-FTIR data demonstrate the formation of PDIBG through ring-opening polymerization of LDIBG.

The synthesized PDIPG homopolymer was characterized using the same procedures applied to PDIBG, and the results were interpreted accordingly. In the ^1^H NMR spectrum (Figure S1D), an apparent triplet (overlapped d) at 1.07 ppm corresponds to the a- and b-labeled C*H*_3_ protons, a multiplet at 2.36 ppm is assigned to the c-labeled C*H* protons, and a doublet at 5.00 ppm corresponds to the d-labeled α-methine protons on the PDIPG backbone. Additionally, the aromatic protons (h) exhibit a signal at 7.36 ppm, confirming the presence of the aromatic ring. The ^13^C NMR spectrum (Figure S1E) shows peaks at 16.99 and 18.69 ppm corresponding to carbons a and b, respectively, a peak at 30.35 ppm for carbon c, and a signal at 76.98 ppm for the α-methine carbon d. Aromatic carbons (h) resonate at 124.46, 128.47, and 128.57 ppm, while the carbonyl carbon (g) of the ester group appears at 168.83 ppm. These results are consistent with the expected PDIPG structure. ATR-FTIR analysis (Figure S1F) exhibits a sharp peak at 1749 cm^–1^, corresponding to the ester carbonyl (C=O) stretching, along with C–H stretching vibrations observed at 2936, 2973, and 2880 cm^–1^. The slight shift of the carbonyl stretching from 1748 cm^–1^ in the LDIPG monomer to 1749 cm^–1^ in PDIPG confirms that ring-opening polymerization was successful.

GPC analysis of PSG homopolymers (Figure 3B and Table 1) shows a single, narrow PDI of 1.45 and no detectable oligomeric species, indicating a controlled polymerization process. The experimentally determined molecular weights are in good agreement with the theoretical *M*_n_ values.

**Figure 3:**
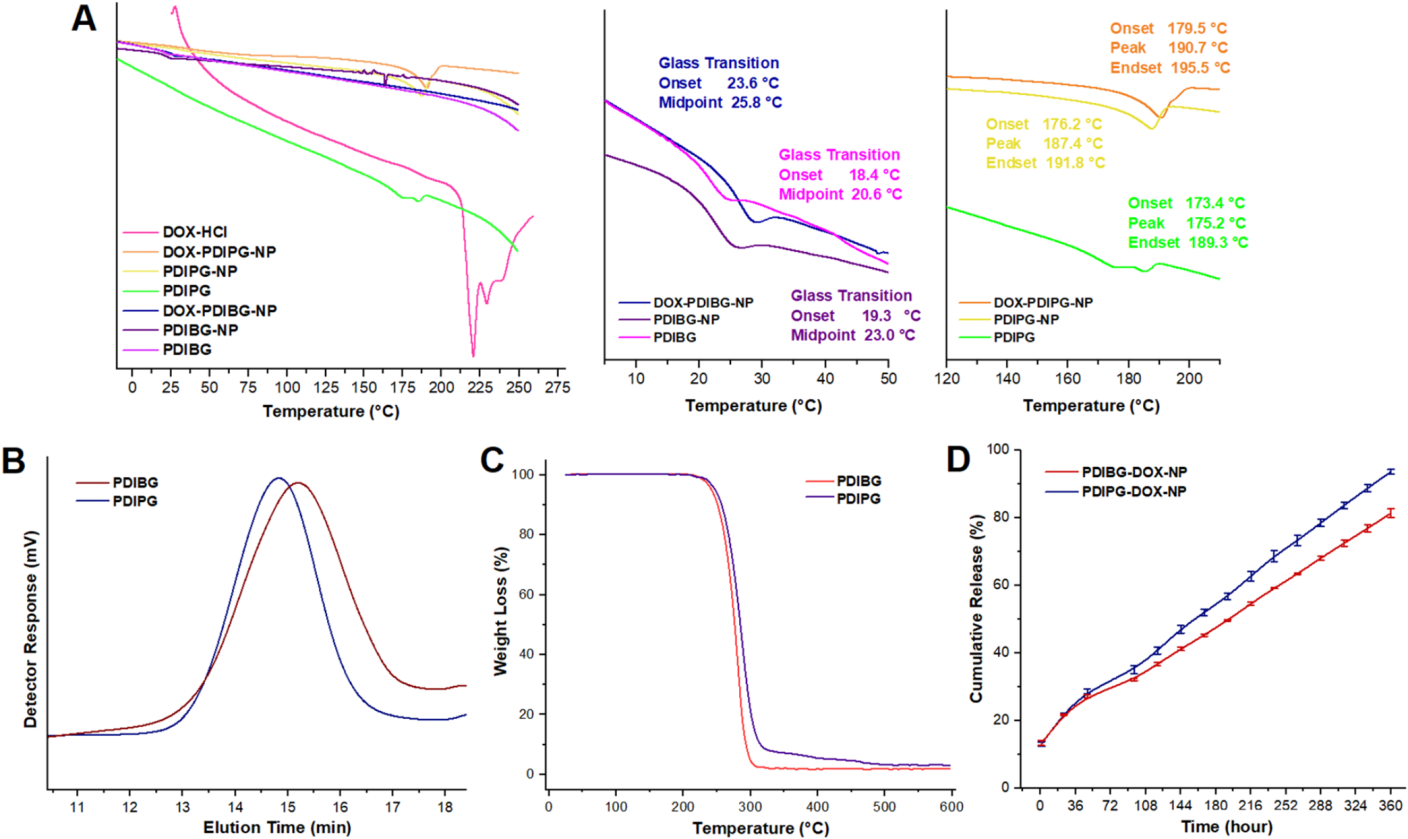
DSC thermograms of PSG homopolymers, DOX, and drug-loaded/drug-free NPs, with magnified views of the glass transition (middle) and melting temperature (right) regions (A), GPC chromotagrams of PSG homopolymers (B), TGA thermograms of PSG homopolymers (C), and DOX release profiles from PSG-NPs (D).

DSC analysis was performed by heating the samples (except DOX-HCl) from −60 to 250 °C at a rate of 10 °C/min, and DOX-HCl was analyzed between 25 and 260 °C at a rate of 10 °C/min. In the thermal characterization of the synthesized homopolymers (Figure 3A), DSC curves revealed distinct thermal behaviors depending on the polymer structure. The PDIBG homopolymer exhibited an amorphous behavior with a glass transition temperature (*T*_g_) of 20.6 °C. For the PDIPG homopolymer, a distinct melting peak (*T*_m_) was recorded at 175.2 °C, with a corresponding enthalpy of melting (Δ*H*) of 11.31 J/g.

Thermogravimetric analysis (TGA) of the PSGs, performed by heating to 600 °C at 10 °C/min (Figure 3C), revealed single-step degradation for both PDIBG and PDIPG, with residual carbon yields of 1.96% and 3.08%, respectively. The onset and endset degradation temperatures were 262.76 and 291.05 °C for PDIBG, and 266.34 and 298.79 °C for PDIPG, indicating similar thermal stability.

Finally, the specific optical rotation values of the polymers were also investigated to assess their stereochemical integrity. The specific optical rotation ([α]_*D*_) values were measured as −81.2° for PDIBG and −69.6° for PDIPG. Although the reduction in optical rotation compared to their respective monomers indicates a degree of partial racemization commonly observed during high-temperature ring-opening polymerization,^53^ our synthesized homopolymers exhibited significantly higher optical rotation magnitudes compared to previous reports (e.g., −6.0° for PDIBG by Cohen-Arazi et al.^61^). This demonstrates that the employed polymerization conditions successfully maintained a high degree of stereoregularity within the polymer chains.

### 3.3 Encapsulation of DOX in PSG-NPs and Optimum NP Formulation

DOX-loaded PSG nanoparticles (PSG-NPs) were prepared by using the single-emulsion solvent evaporation method (Figure 4). To optimize the polymer amount for NP production, the effects of three different amounts of PDIBG or PDIPG (50, 70, and 100 mg) on particle size, polydispersity index (PDI), NP yield, and drug-loading efficiency were evaluated. As summarized in Table 2, increasing the polymer amount generally resulted in larger particle sizes and higher PDI values for both polymer systems. In PDIPG formulations, NP yield and EE increased with increasing polymer amount, whereas PDIBG formulations showed the highest NP yield at 70 mg of polymer.

**Figure 4:**
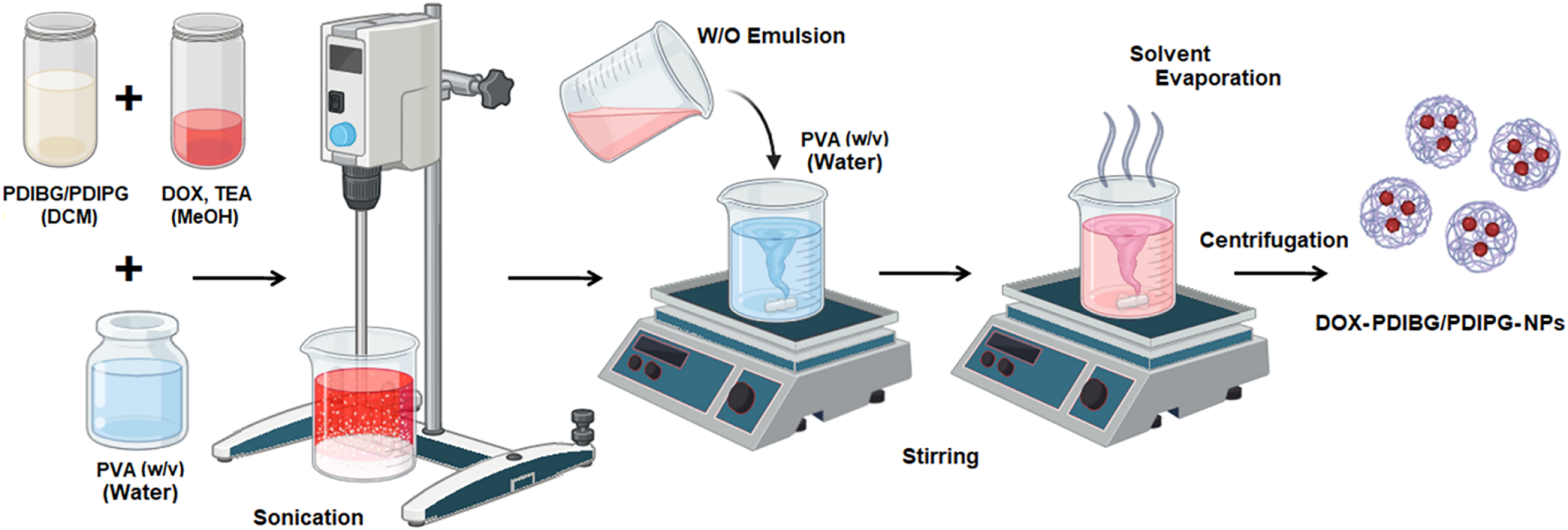
Fabrication of DOX-loaded PSG-NPs via a single-emulsion solvent evaporation method.

**Table 2:** Effects of Different Polymer:Drug Concentrations on the Properties of DOX-PSG-NPs

polymer	amount of polymer (mg)	amount of initial DOX (μg)	size (*Z*-ave) (nm ± SD)	PDI ± SD	NP yield (%)	EE (%)	dltheoric (%)	dlexp (%)
PDIBG	50	521.6 ± 84.0	239 ± 4	0.05 ± 0.04	58.8	59.8	1.03	0.53
PDIPG	50	551.4 ± 33.3	229 ± 4	0.06 ± 0.02	72.2	69.9	1.09	0.54
PDIBG	70	702.1 ± 67.1	253 ± 7	0.06 ± 0.02	83.3	58.3	0.99	0.49
PDIPG	70	561.5 ± 30.4	253 ± 7	0.08 ± 0.04	79.3	73.9	0.80	0.53
PDIBG	100	695.1 ± 112.7	304 ± 20	0.16 ± 0.09	68.9	68.9	0.69	0.70
PDIPG	100	583.3 ± 35.2	294 ± 12	0.15 ± 0.02	83.3	74.5	0.58	0.52

Using 100 mg of polymer resulted in the highest EE values, reaching 68.9% for DOX-loaded PDIBG-NPs and 74.5% for DOX-loaded PDIPG-NPs. However, these formulations also exhibited the largest particle sizes, measuring 304 ± 20 nm and 294 ± 12 nm, respectively, along with relatively higher PDI values of 0.16 ± 0.09 and 0.15 ± 0.02, indicating lower size uniformity.

At 70 mg polymer, DOX-PDIBG-NPs showed a particle size of 253 ± 7 nm, a PDI of 0.06 ± 0.02, an NP yield of 83.3%, and an EE of 58.3%. Similarly, DOX-PDIPG-NPs exhibited a particle size of 253 ± 7 nm, a PDI of 0.08 ± 0.04, an NP yield of 79.3%, and an EE of 73.9%. These formulations demonstrated a favorable balance between particle size, colloidal uniformity, NP yield, and encapsulation performance.

When 50 mg of polymer was used, DOX-PDIBG-NPs and DOX-PDIPG-NPs exhibited smaller particle sizes of 239 ± 4 nm and 229 ± 4 nm, respectively, with low PDI values of 0.05 ± 0.04 and 0.06 ± 0.02. The corresponding EE values were 59.8% for PDIBG-NPs and 69.9% for PDIPG-NPs. Although these formulations showed the smallest particle sizes and narrow size distributions, their NP yields were lower compared with the 70 mg formulations. Overall, the results suggest that 70 mg of polymer provides the most suitable balance between particle size, PDI, NP yield, and EE. Therefore, this polymer amount was selected for all subsequent NP preparations.

Overall, DOX was encapsulated into PDIPG-NPs with higher efficiency than into PDIBG-NPs. This difference may be attributed to the distinct structural properties of the polymers, particularly their different degrees of crystallinity. PDIBG exhibits a predominantly amorphous character, whereas PDIPG possesses a semicrystalline structure. The more ordered semicrystalline arrangement of PDIPG may promote tighter polymer chain packing during nanoparticle formation, thereby enhancing drug–polymer interactions and reducing drug diffusion from the nanoparticle matrix. In contrast, the more disordered amorphous structure of PDIBG may lead to a less compact nanoparticle core and comparatively lower EE value. Although the exact mechanism has not yet been fully elucidated, similar effects of polymer architecture and structural order on drug encapsulation have been reported for PEG-based PLGA nanoparticles. In particular, it has been suggested^62^ that the more disordered structure of diblock PEG–PLGA copolymers may hinder interactions between drug/protein molecules and PLGA chains within the polymer matrix.

In our previous study related to PLGA polymer,^12^ higher polymer amounts (70 mg) resulted in increased NP yields; however, redispersion of the nanoparticles after centrifugation and washing was more difficult compared to lower polymer amounts. Furthermore, increasing the polymer amount from 50 to 100 mg resulted in a decrease in drug-loading capacity (DL%). Similarly, Pieper et al.^63^ demonstrated a generally inverse relationship between NP yield and DL% when using pure PLA and PLGA (100 and 50 mg, respectively) and PEG-based PLGA (50 mg). In NPs composed of pure PLA/PLGA, lower NP yields were associated with higher DL, whereas in PEG-based systems, higher NP yields corresponded to lower DL. In contrast, in the present study, the DL% values showed relatively minor variations despite changes in NP yield with increasing polymer amounts in DOX-loaded PDIBG and PDIPG-NPs. This behavior indicates a more stable drug-loading profile compared to PLA/PLGA- or PEG-based PLGA systems and suggests strong drug–polymer interactions within the PDIBG and PDIPG nanoparticle systems. Moreover, although the 100 mg formulations exhibited comparable or higher EE% values, the increase in particle size and PDI indicated reduced colloidal uniformity. Therefore, considering the desired particle size, low PDI, high NP yield, and satisfactory EE% values together, 70 mg of polymer was determined to be the most suitable ratio for the preparation of PSG-NPs. All subsequent experiments were conducted using nanoparticles prepared with this polymer amount.

Following the optimization of DOX-loaded PSG-NPs, the thermal characterization of the nanoparticles was conducted to evaluate the physical state of the encapsulated drug and its interaction with the polymer matrices (Figure 3A). When the formulated nanoparticles were evaluated, the thermal properties of the base polymers were found to be slightly altered upon drug encapsulation.

Specifically, the *T*_g_ of blank PDIBG-NPs was measured at 23.0 °C, and the incorporation of DOX led to a slight upward shift to 25.8 °C. This behavior aligns with the findings of Misiak et al.,^6^ who reported that DOX incorporation into polymeric matrices restricts chain mobility via strong intermolecular interactions, thereby elevating the *T*_g_. Similarly, for the blank PDIPG-NPs, a melting peak was observed at 187.4 °C, which shifted to 190.7 °C upon DOX encapsulation. This is consistent with previous studies^31^ demonstrating that DOX undergoes a transition from a crystalline to an amorphous state upon encapsulation, and its strong interaction with the polymer matrix can induce a slight positive shift in the polymer’s melting peak. To further validate this structural reorganization, Δ*H* values for blank PDIPG-NPs and DOX-PDIPG-NPs were evaluated and found to be 13.8 and 19.2 J/g, respectively (compared to 11.3 J/g for the PDIPG). The increase in enthalpy suggests altered thermal behavior and structural reorganization within the polymer matrix, likely arising from strong drug–polymer interactions and changes in molecular packing, which consequently requires higher thermal energy to melt.

### 3.4 Drug Release Study

The drug release profiles of DOX-loaded PSG-NPs over 360 h at 37 °C in PBS (pH 7.4) are presented in Figure 3D. Specifically, 93.6% of DOX was released from PDIPG-NPs, while 81.4% was released from PDIBG-NPs. At the end of the release period, a greater proportion of DOX was released from PSG-NPs compared to previously reported PLGA-NPs, which showed cumulative release values of 76.7% (PLGA 75:25) and 76.7% (PLGA 50:50). This enhanced release behavior may be attributed to differences in molecular weight or polymer structure (branched isobutyl and isopropyl side chains), which could increase free volume within the matrix. Such structural features may facilitate water uptake and drug diffusion, leading to faster release kinetics. In agreement with this, Choi et al.^30^ reported that lower molecular weight PLGA exhibits faster drug release compared to higher molecular weight systems, suggesting that polymer chain characteristics can significantly affect release kinetics.

### 3.5 Lyophilization Study of DOX-PSG-NPs

#### 3.5.1 Effect of Different Freezing Temperatures on the Lyophilized NP Properties

Lyophilization of the prepared DOX-PSG-NPs (Figure 5) was performed after freezing at two different temperatures, −20 °C and −50 °C. Among all tested conditions, 5% sucrose demonstrated the most effective cryoprotective performance for both PDIBG- and PDIPG-based nanoparticles, particularly at −50 °C, yielding the smallest particle size and lowest PDI values, while mannitol failed to provide adequate stabilization under all conditions. (Figure 5A,B). Detailed DLS size and analysis of the rehydrated lyophilized NPs, restored to volumes similar to their initial state, are summarized in Figures S2–S5 and Tables S1 and S2. In the absence of added sugar, lyophilization caused significant NP aggregation at both freezing temperatures, accompanied by a substantial increase in particle size and PDI (Tables S1 and S2; Entry 2). Aggregation was more pronounced in samples frozen at −20 °C compared to −50 °C. The addition of 1% of all sugars (Tables S1 and S2; Entries 3–5) or 5–10% mannitol (Tables S1 and S2; Entries 6 and 9) did not sufficiently improve the physical stability of the NPs. In contrast, glucose and sucrose at 5% (Tables S1 and S2; Entries 7 and 8) or 10% (Tables S1 and S2; Entries 10 and 11) effectively preserved particle size and PDI, demonstrating superior cryoprotective performance. Overall trends were similar at both freezing temperatures, although slightly better preservation was observed at −50 °C in some formulations. Considering practical aspects, −20 °C was selected for subsequent lyophilization experiments due to its convenience and reproducibility.

**Figure 5:**
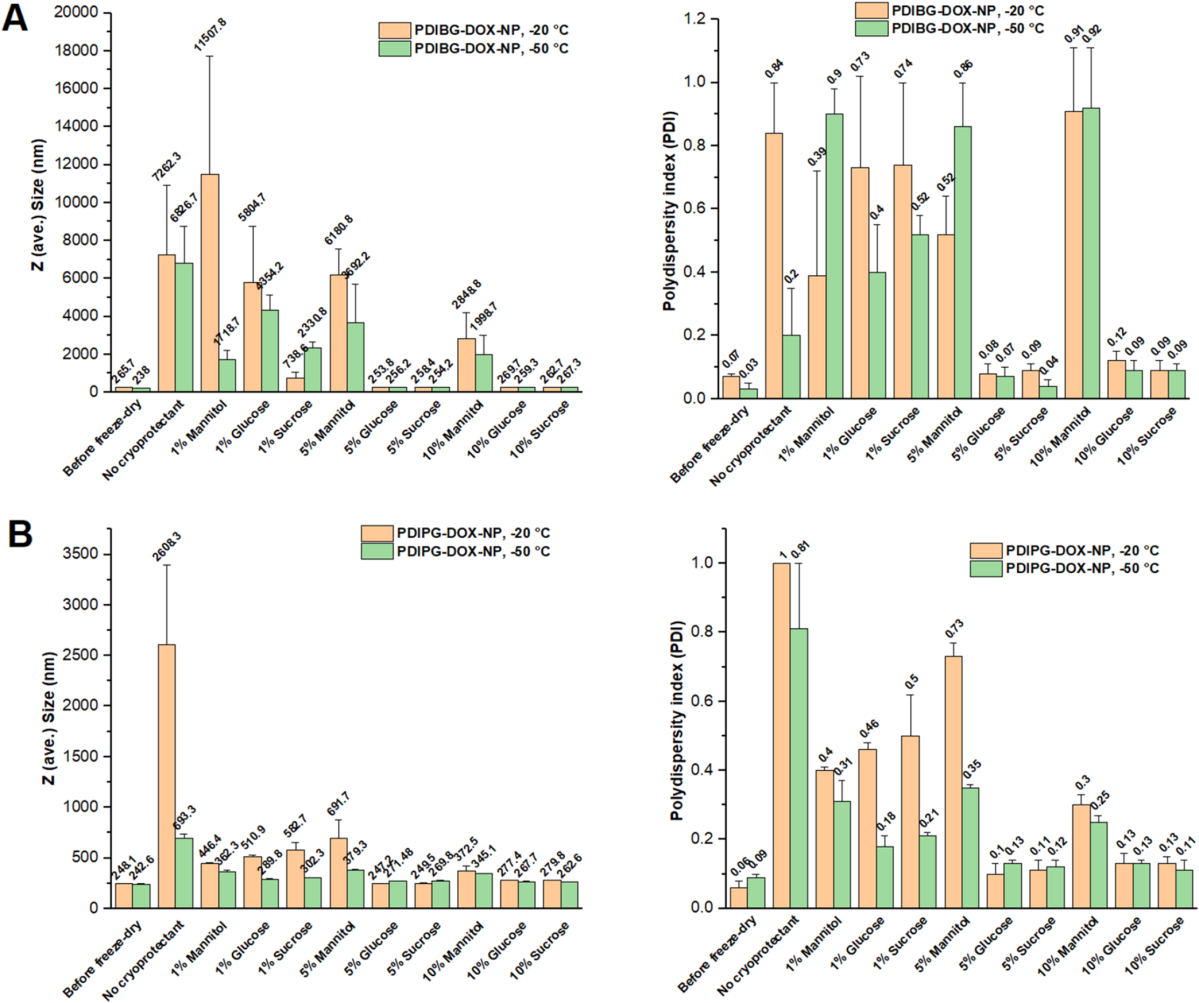
Size and PDI values of DOX-PSG-NPs particles lyophilized at different freezing temperatures.

#### 3.5.2 Effect of Different NP Concentrations on Lyophilized NP Properties

To evaluate the effect of nanoparticle concentration on physical stability during lyophilization, suspensions of DOX-PSG-NPs at 50, 70, and 90 mg/mL were mixed with final 5% or 10% glucose or sucrose solutions and lyophilized at −20 °C. After reconstitution, for DOX-loaded PDIBG-NPs, both 5% and 10% glucose or sucrose effectively preserved particle stability (size and PDI) at 50 mg/mL (Figure 6A and Table S3, Entries 3–6), preventing aggregation (Figures S6–S8). At 70 mg/mL, stability was maintained only with 10% glucose (Figure 6A and Table S3, Entry 5), whereas at 90 mg/mL, only 10% sucrose (Figure 6A and Table S3, Entry 6) remained effective. In contrast, DOX-loaded PDIPG-NPs retained physical stability across all tested concentrations (50–90 mg/mL) when either 5% or 10% glucose or sucrose was used, with no significant aggregation observed (Figures 6B and S9–S11 and Table S4, Entries 3–6). Overall, DOX-PDIBG-NPs maintained particle integrity at 50 mg/mL under all cryoprotectant conditions (Figure 6A); however, stability at higher concentrations was dependent on cryoprotectant type and concentration. In contrast, DOX-PDIPG-NPs remained stable across the entire concentration range (50–90 mg/mL), regardless of cryoprotectant type or concentration (Figure 6B). These results indicate that PDIPG-NPs exhibit higher robustness during lyophilization, while appropriate concentrations of glucose or sucrose act as effective cryoprotectants, particularly at higher nanoparticle concentrations.

**Figure 6:**
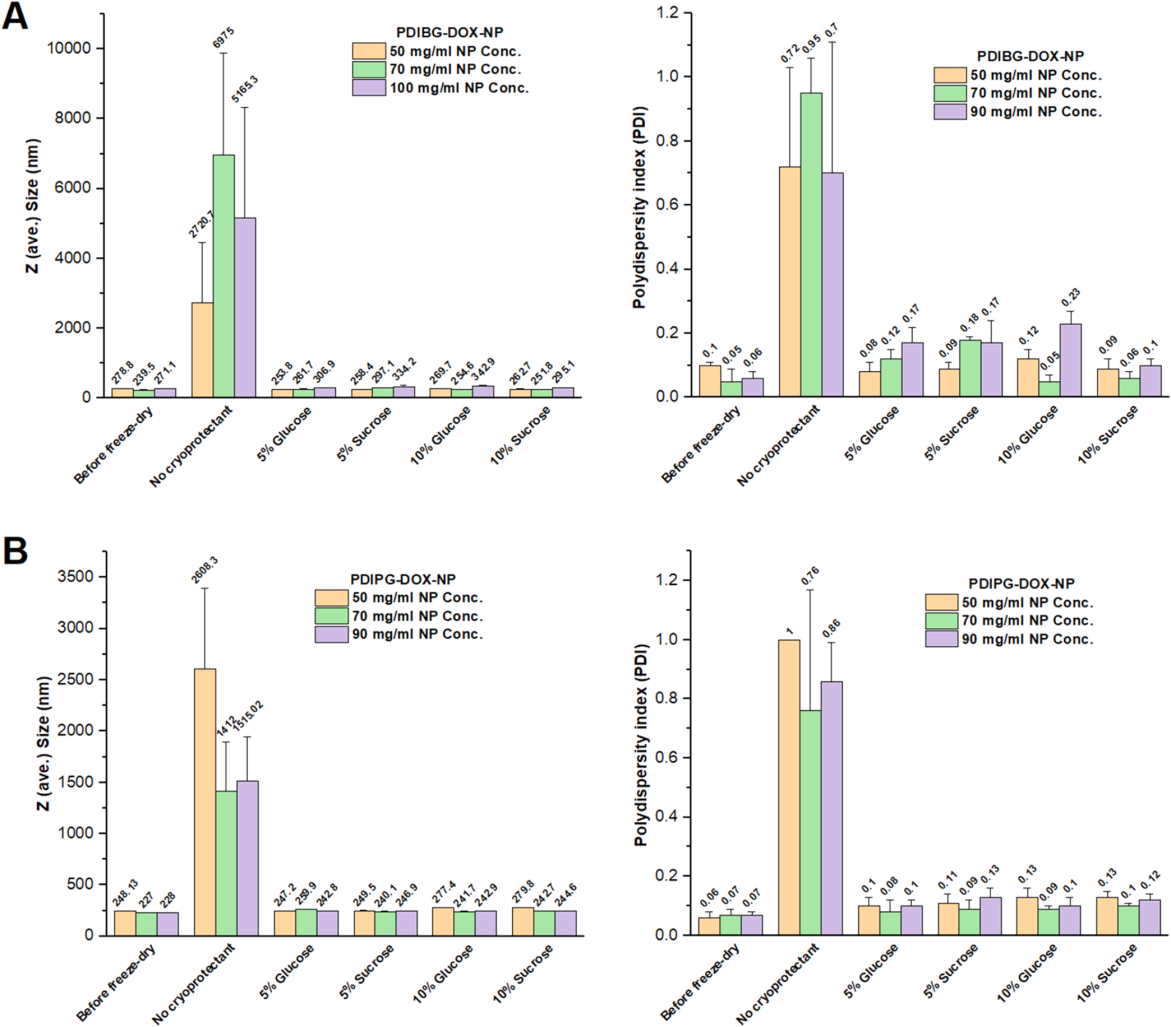
Size and PDI values of lyophilized DOX-PSG-NPs at different NP:cryo concentrations.

### 3.6 Physical Stability Study of DOX-PSG-NPs

Following extensive optimization of the lyophilization process, including variations in freezing temperature, sugar type and concentration, and NP concentration, the optimal conditions for DOX-loaded PSG-NPs were established. NP formulations at 50 mg/mL were combined with 5% or 10% glucose or sucrose, frozen at −20 °C, and lyophilized overnight. The lyophilized NPs were subsequently stored at 4 °C (Figures 7 and S12–S15) and −20 °C (Figures 8 and S16–S19) for up to 2 months.

**Figure 7:**
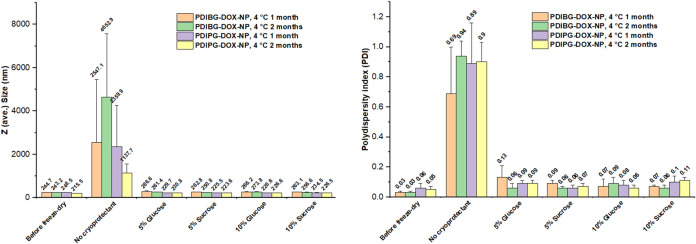
Storage of lyophilized DOX-PSG-NPs at 4 °C for 2 months.

**Figure 8:**
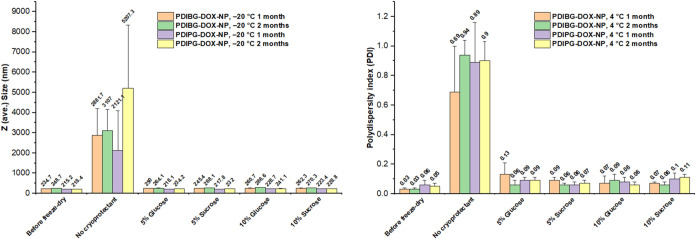
Storage of lyophilized DOX-PSG-NPs at −20 °C for 2 months.

Formulations without added sugar (Figures 7 and 8 and Tables S5 and S6; Entries 3 and 4) exhibited a significant increase in particle size and PDI beginning from the first month of storage, accompanied by visible aggregation compared with their initial particle sizes (Figures 7 and 8 and Tables S5 and S6; Entries 1 and 2). In contrast, formulations containing 5% or 10% glucose or sucrose (Figures 7 and 8 and Tables S5 and S6; Entries 5–12) maintained NP stability, enabling DOX-loaded PSG-NPs to retain colloidal stability for up to 2 months at both storage temperatures.

Consistent with the DLS results, SEM analysis (Figure 9) of lyophilized DOX-PDIPG-NPs, used as a representative example and stored at −20 °C for 2 months, demonstrated that particles without sugar formed aggregates, whereas particles containing glucose or sucrose (5% or 10%) retained their spherical morphology without noticeable clustering.

**Figure 9:**
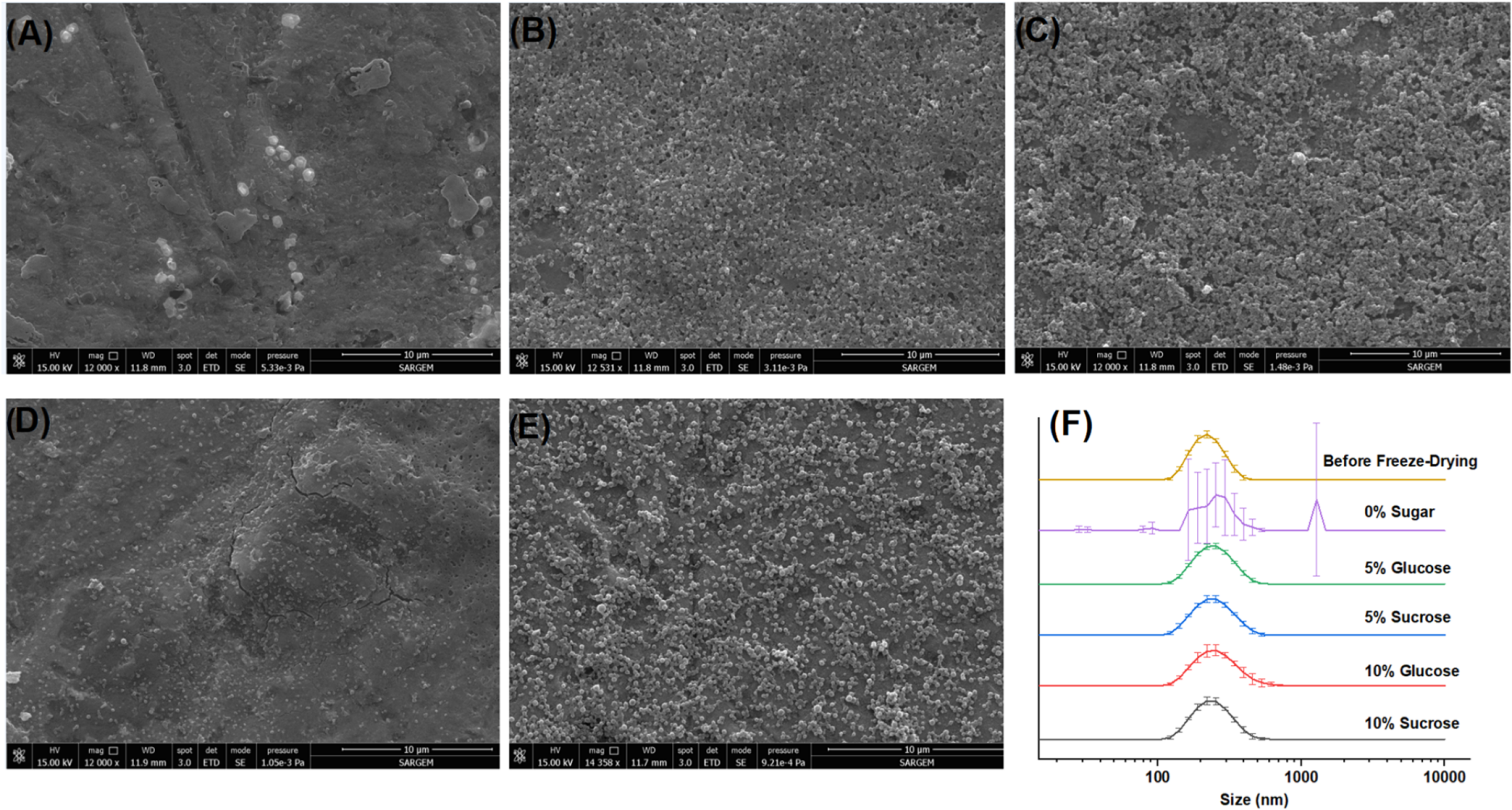
SEM microphotographs of lyophilized PDIPG-DOX-NPs in sugar-free (A), 5% glucose-containing (B), 5% sucrose-containing (C), 10% glucose-containing (D), 10% sucrose-containing (E) formulations stored at −20 °C for 2 months. (Scale bar: 10 μm), DLS graph of the same PDIPG-DOX-NPs (F).

As previously reported,^12^ the addition of 5% or 10% sugars during lyophilization preserved the stability of DOX-loaded PLGA 50:50 and 75:25 NPs during storage at −20 °C for 2 months. However, at 4 °C, both sugar concentrations were effective for PLGA 50:50 NPs, whereas only 10% sugar maintained the stability of PLGA 75:25 NPs. When these findings are considered alongside reports in the literature, the results indicate that glucose and sucrose effectively function as cryoprotectants by maintaining the post-storage stability of PLGA and PSG-NPs during long-term storage.

### 3.7 Hydrolytic Degradation Studies of PSG-NPs

The hydrolytic degradation behavior of PSG-NPs at different time points was evaluated (Figure 10). In previous studies,^41^ it has been reported that the PDIBG homopolymer is highly hydrophobic and resistant to hydrolysis due to the diisobutyl side chains in its structure. As shown in Table 3, when the average molecular weight (*M*_n_) of PDIBG-NPs was assessed after 35 days, a slight decrease was observed (12,220 to 11,450, day 0–35), and the *M*_n_ % loss value was reported as 6.3. In comparison to PDIBG-NPs, it was observed that the hydrolytic degradation of PDIPG-NPs was relatively faster (9290 to 8160, day 0–35), with the *M*_n_ % loss value for PDIPG-NPs resulting in 12.2. It is suggested that the reason PDIBG-NPs exhibit a slower degradation behavior compared to PDIPG-NPs is due to the higher molecular weight of the PDIBG homopolymer; the degradation behavior of polymers with higher molecular weights has also been explained in the literature as being due to the fact that their longer chains require a longer time to degrade.^62,64^ Furthermore, when the release behavior of DOX-PSG-NPs is compared, the fact that PDIPG-NPs exhibit a faster drug release profile (93.6% vs 81.4%) is closely related to their degradation behavior.^65^

**Figure 10:**
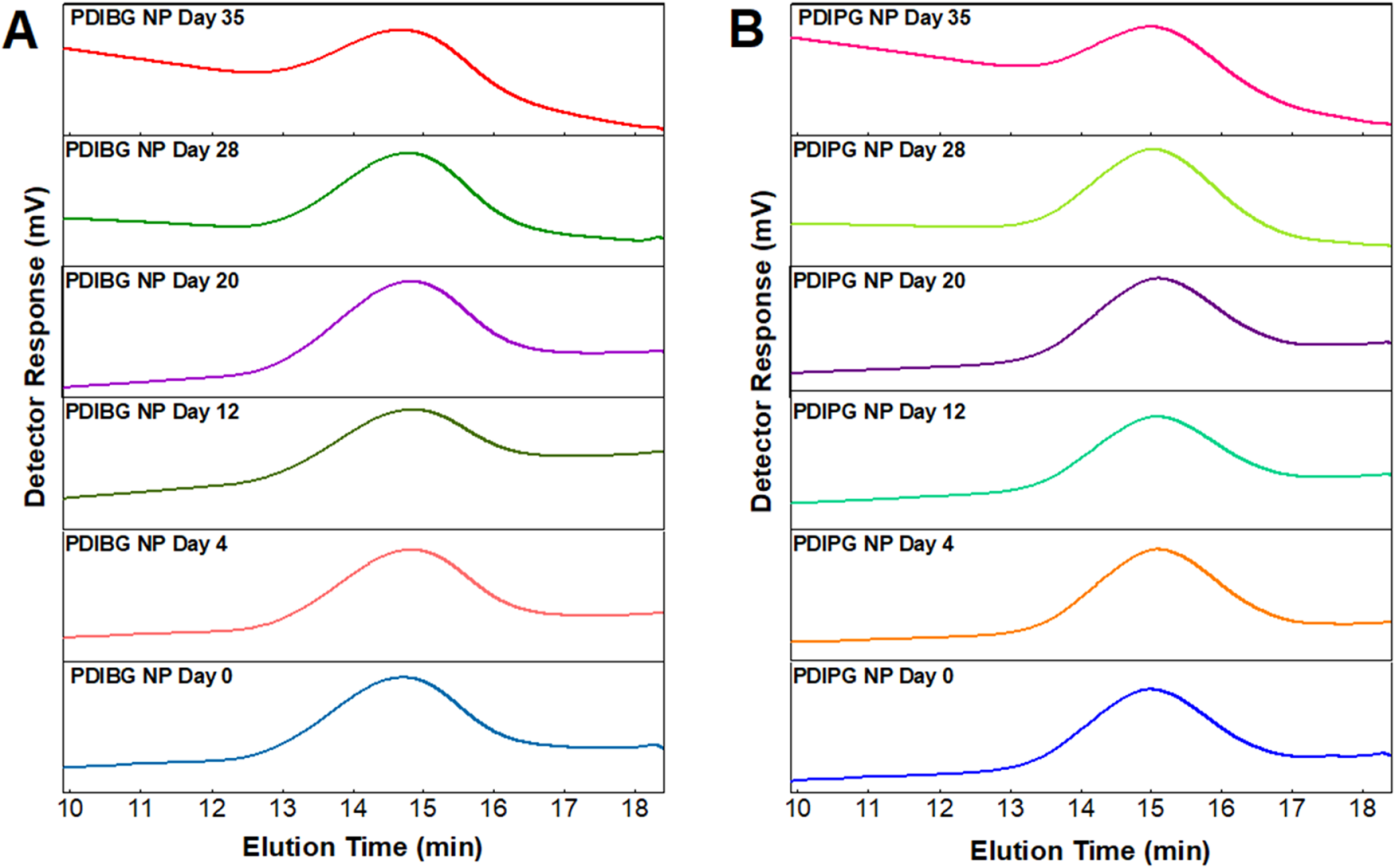
Hydrolytic degradation curves of PDIBG-NPs (A) and PDIPG-NPs (B) at different time points, evaluated by GPC.

**Table 3:** Hydrolytic Degradation of PSG-NPs^a^

time	name	*M*_w_	*M*_n_	*M*_w_/*M*_n_	*M*_n_ % loss
day 0	PDIBG-NP	19,180	12,220	1.57	
	PDIPG-NP	13,560	9290	1.46	
day 4	PDIBG-NP	16,930	11,720	1.44	4.1
	PDIPG-NP	12,070	8630	1.40	7.1
day 12	PDIBG-NP	17,630	11,710	1.51	4.2
	PDIPG-NP	12,350	8610	1.44	7.3
day 20	PDIBG-NP	17,060	11,730	1.45	4.0
	PDIPG-NP	12,230	8540	1.43	8.1
day 28	PDIBG-NP	17,513	11,391	1.53	6.8
	PDIPG-NP	12,720	8160	1.49	12.2
day 35	PDIBG-NP	17,100	11,450	1.49	6.3
	PDIPG-NP	12,190	8160	1.49	12.2

a *M*_n_ % loss was calculated using the equation “(*M*_n,0_ – *M*_n,t_)/(*M*_n,0_) × 100”.

## 4 Conclusions

In this study, PDIBG and PDIPG homopolymers, which are highly suitable for nanoparticle production in controlled drug-delivery systems, were successfully synthesized from PSG. The synthesized PSG homopolymers were subsequently used to prepare DOX-loaded nanoformulations via the single-emulsion solvent evaporation method, and long-term-storable powder forms of these nanoparticles were obtained through lyophilization.

As the initial step of polymer synthesis, the corresponding hydroxy acids, IBHA and IPHA, were synthesized from l-leucine and l-valine amino acids using the diazotization method. These hydroxy acids were subsequently refluxed in toluene in the presence of *p*-toluenesulfonic acid monohydrate (PTSA·H_2_O) to obtain the substituted glycolide monomers LDIBG and LDIPG. Subsequently, ring-opening polymerization of the resulting monomers was then performed in bulk using benzyl alcohol as the initiator and Sn­(Oct)_2_ as the catalyst. Molecular weight analyses of PDIBG and PDIPG showed good agreement in NMR spectroscopy and GPC results, yielding *M*_n_ values of 12,350 and 10,400 g/mol, respectively, with a narrow PDI of 1.45. Thermal analysis revealed that PDIBG exhibited a *T*_g_ of 20.62 °C, while PDIPG showed a melting temperature (*T*_m_) of 175.22 °C. Upon heating to 600 °C, PDIBG and PDIPG displayed char yields of 1.96% and 3.08%, respectively. The decomposition onset (*T*_onset_) and endset (*T*_endset_) temperatures were determined to be 262.76 and 291.05 °C for PDIBG, and 266.34 and 298.79 °C for PDIPG.

The effects of increasing polymer amounts (50, 70, and 100 mg) on particle size, PDI, nanoparticle yield, and drug-loading capacity of PSG-NPs prepared by the single-emulsion solvent evaporation method were systematically evaluated. Based on these results, 70 mg of polymer was identified as the optimal amount for producing the most suitable PSG-DOX nanoformulation. To obtain stable powder forms of the PSG-DOX-NPs, lyophilization was employed, and appropriate cryoprotectants were added before freeze-drying to prevent nanoparticle aggregation. Furthermore, nanoparticle concentration was identified as a key determinant of lyophilization performance. DOX-PDIBG-NPs exhibit behavior dependent on cryoprotectant type and concentration; they maintain their physicochemical integrity at 50 mg/mL under all conditions, but require specific cryoprotectant systems at higher concentrations. On the other hand, DOX-PDIPG-NPs demonstrate consistent stability across the entire range examined (50–90 mg/mL), regardless of cryoprotectant type and concentration.

The effects of different sugar types and concentrations, as well as freezing temperatures, on the physicochemical properties of lyophilized nanoparticles were systematically evaluated. It was observed that the addition of glucose or sucrose at concentrations of 5% and 10%, combined with a freezing temperature of −20 °C, effectively preserved particle size and size distribution following long-term storage. Under these optimized conditions, lyophilized PDIBG/PDIPG-DOX-NP powders remained stable for up to 2 months when stored at both 4 °C and −20 °C. The comprehensive lyophilization study conducted herein provides valuable guidance for defining key processing parameters, including cryoprotectant selection and freezing temperature, for the long-term storage of PSG-based nanoparticles. These findings demonstrate that, upon validation of their clinical applicability, PSG-NPs can be formulated into solid forms that maintain acceptable postreconstitution particle size, PDI, and redispersibility during storage, supporting their potential for improved storage stability and practical pharmaceutical use.

## Supplementary Material



## Data Availability

The data that support the findings of this study are available in the Supporting Information of this article.
