# Supplementary material for: Doxorubicin-Loaded Poly(Substituted Glycolide)-Based Nanoparticles for Long-Term Storage
Source: ACS Omega. 2026 Jun 6;11(24):35488–502. doi: 10.1021/acsomega.6c01126 (PMC13294889; doi:10.1021/acsomega.6c01126)
Supplement: Supplementary file 1 [file ao6c01126_si_001.pdf]

Supporting Information

Doxorubicin-Loaded Poly(Substituted Glycolide) Based Nanoparticles for Long-Term Storage

Tuğba Koldankaya[1], Mehmet Onur Arıcan[1], Olcay Mert[1,2], Serap Mert[1,2,3] *

[1.] *Department of Polymer Science and Technology, Kocaeli University, 41001, Kocaeli, Türkiye*

[2.] *Department of Chemistry*, Faculty of Arts and Sciences, *Kocaeli University*, 41001, *Kocaeli*, Türkiye

[3.] *Center for Stem Cell and Gene Therapies Res. and Pract., Kocaeli University, 41001 Kocaeli, Türkiye*

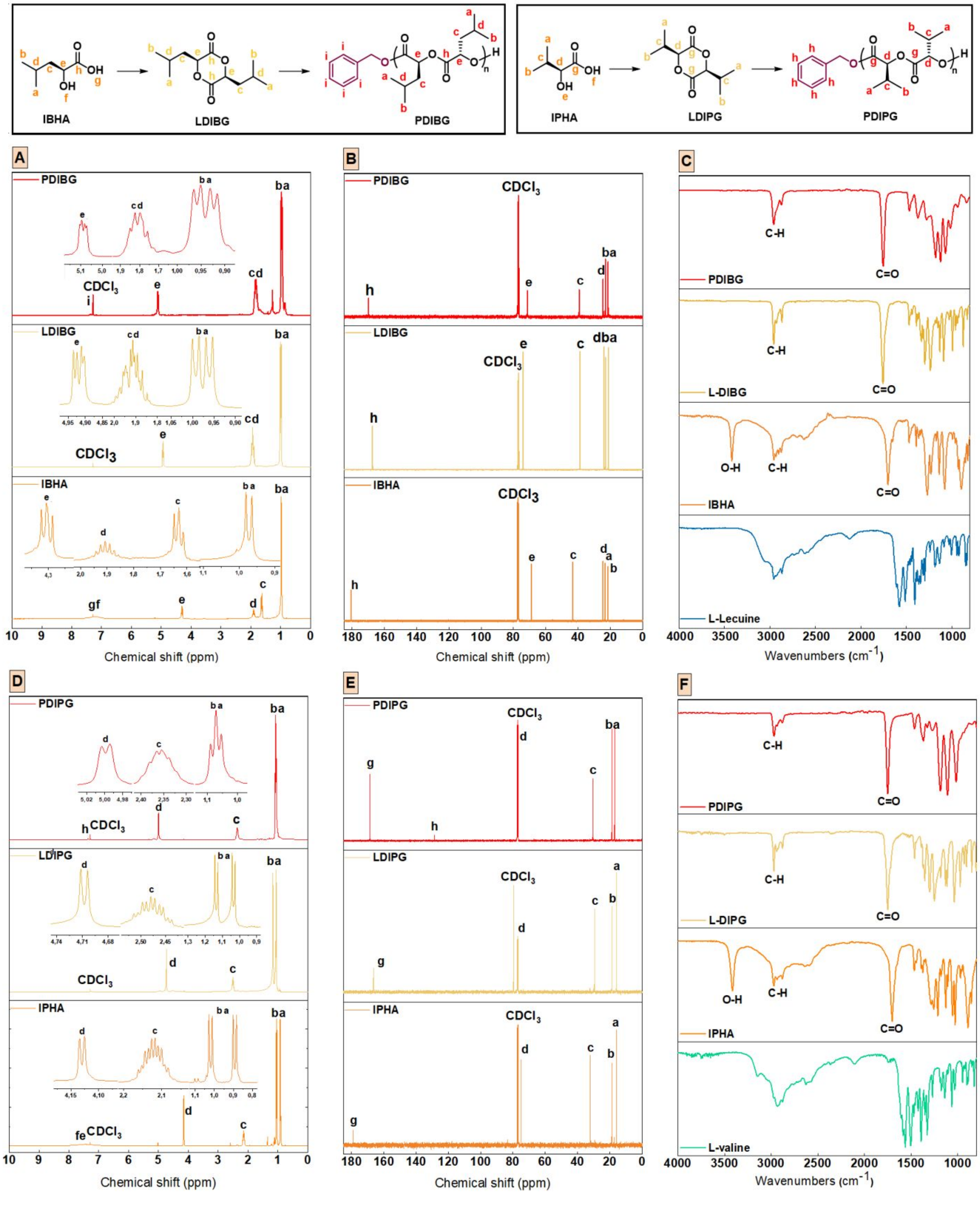

Figure S1. Structural characterization of the synthesized molecules: ^{1}H-NMR spectrum (A), ^{13}C-NMR spectrum (B), ATR FT-IR spectrum (C) of PDIBG, LDIBG, and IBHA; ^{1}H-NMR spectrum (D), ^{13}C-NMR spectrum (E), ATR FT-IR spectrum (F) of PDIPG, LDIPG and IPHA.

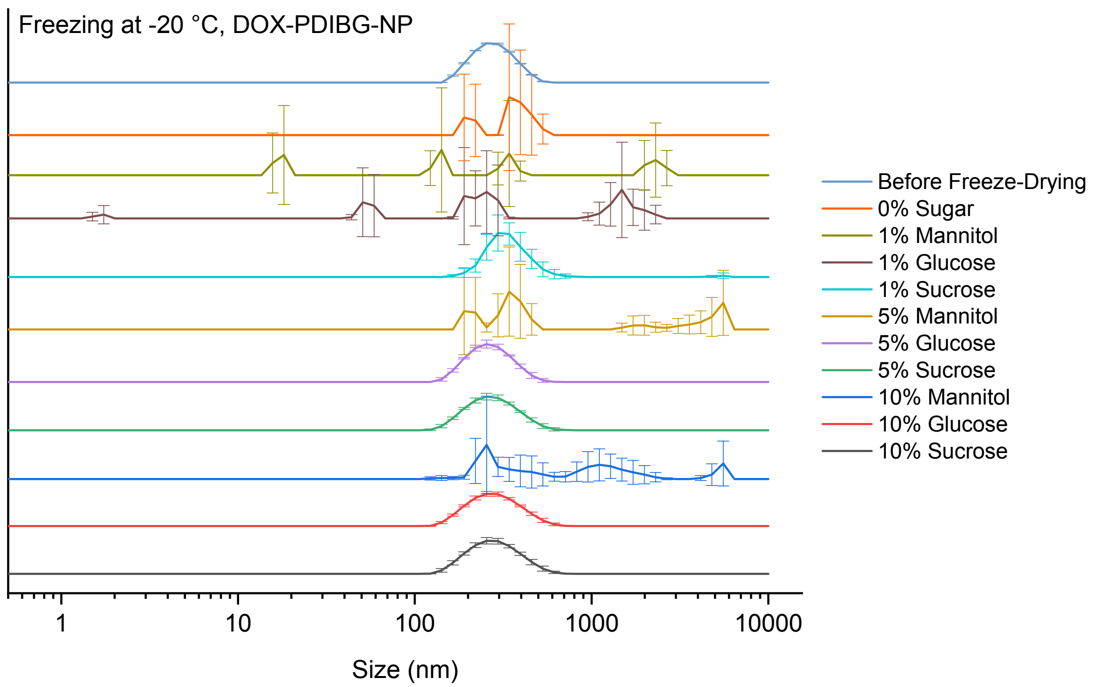

Figure S2. Lyophilized at a freezing temperature of -20 °C, DOX-PDIBG-NPs.

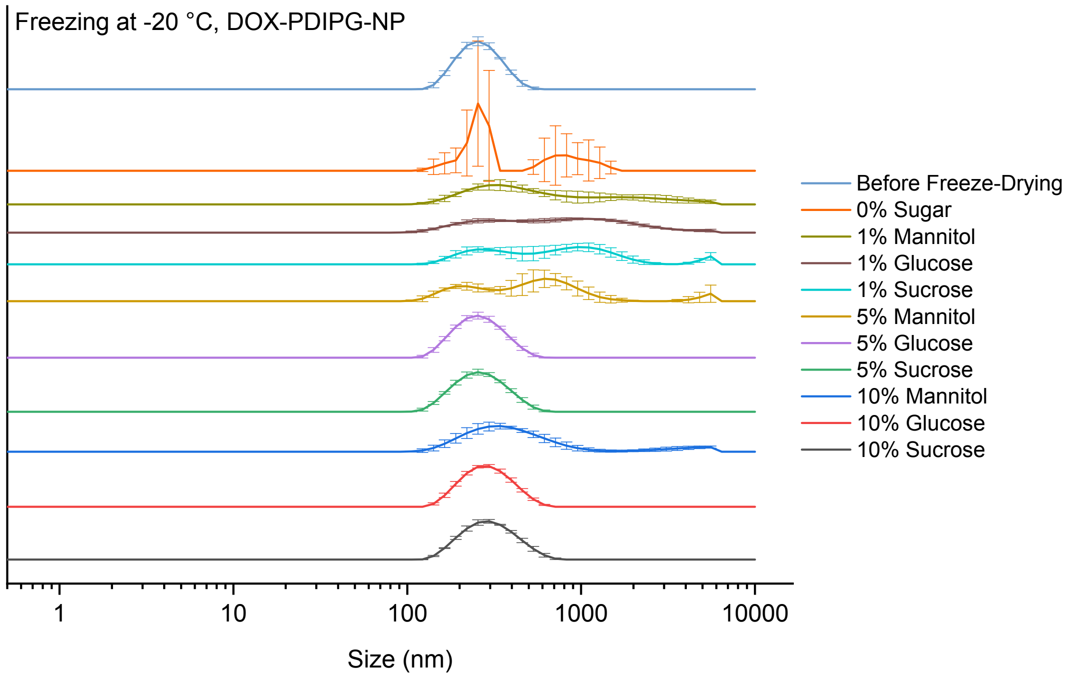

Figure S3. Lyophilized at a freezing temperature of -20 °C, DOX-PDIPG-NPs.

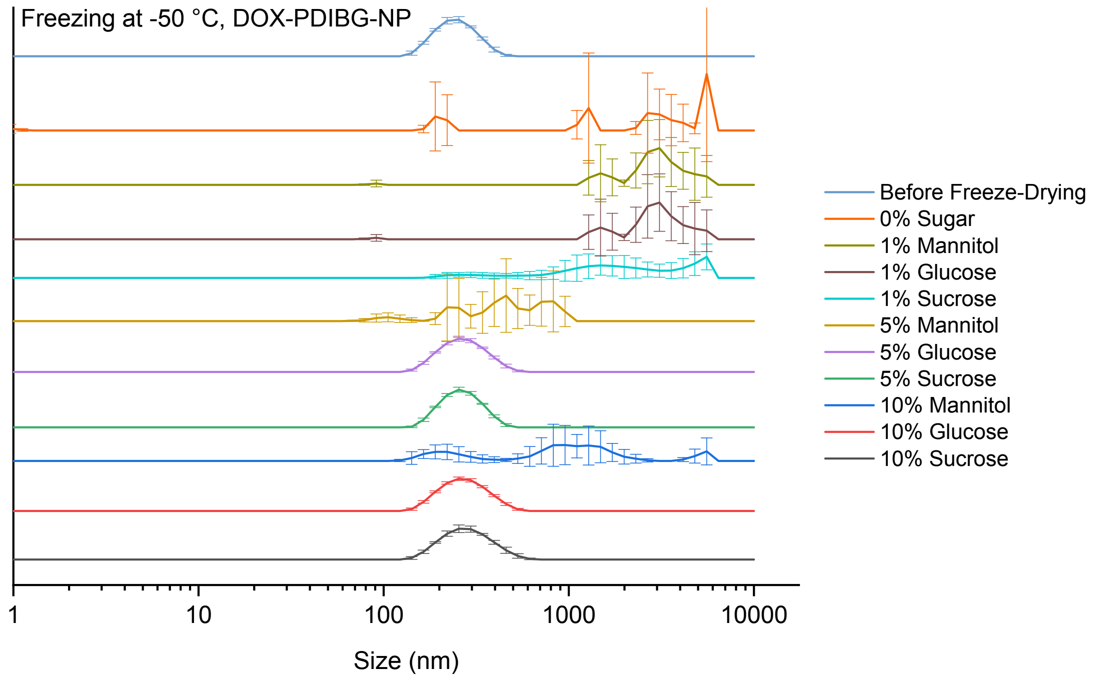

Figure S4. Lyophilized at a freezing temperature of -50 °C, DOX-PDIBG-NPs.

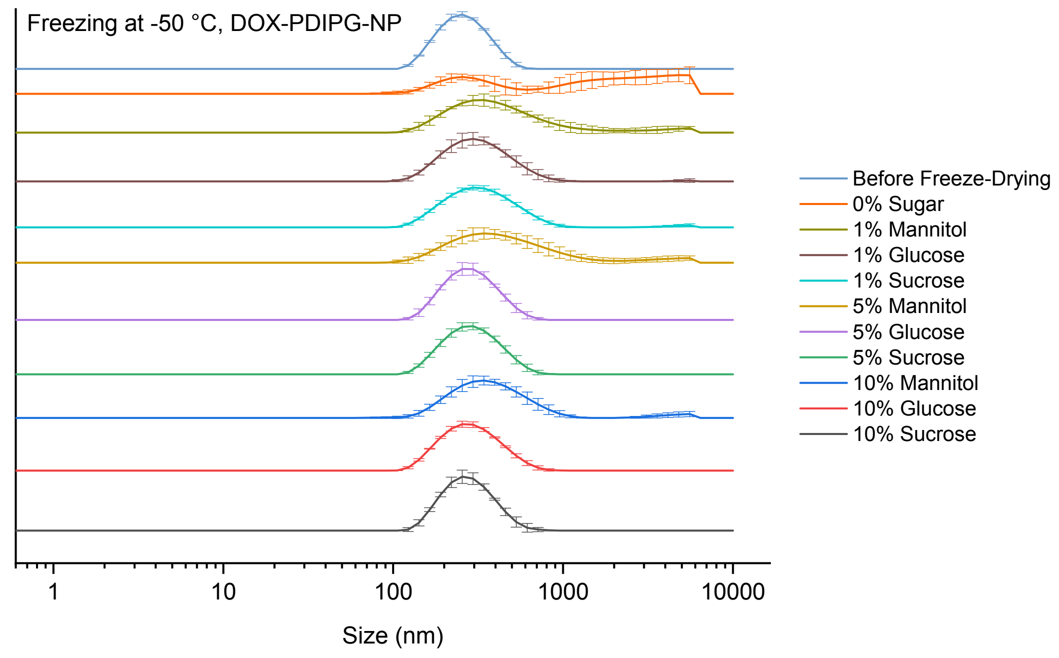

Figure S5. Lyophilized at a freezing temperature of -50 °C, DOX-PDIPG-NPs.

Table S1. DOX-PDIBG-NP particles lyophilized at different freezing temperatures.

DOX-PDIBG-NP		***− 20 °C***			***− 50 °C***		
Entry	*Conditions*	*Size (Z.ave) nm ± SD*	*PDI ± SD*	*Aggr*	*Size (Z.ave) nm ± SD*	*PDI ± SD*	*Aggr*
1	Before freeze-dry	266 ± 1	0.07 ± 0.01	-	238 ± 1	0.03 ± 0.02	-
2	No cryoprotectant	7262 ± 3644	0.84 ± 0.16	+	6827 ± 1916	0.20 ± 0.15	+
3	1% Mannitol	11508 ± 6199	0.39 ± 0.33	+	1719 ± 474	0.90 ± 0.08	+
4	1% Glucose	5805 ± 2926	0.73 ± 0.29	+	4354 ± 764	0.40 ± 0.15	+
5	1% Sucrose	739 ± 306	0.74 ± 0.26	+	2331 ± 345	0.52 ± 0.06	+
6	5% Mannitol	6181 ± 1369	0.52 ± 0.12	+	3692 ± 2020	0.86 ± 0.14	+
7	5% Glucose	254 ± 3	0.08 ± 0.03	-	256 ± 3	0.07 ± 0.03	-
8	5% Sucrose	258 ± 3	0.09 ± 0.02	-	254 ± 2	0.04 ± 0.02	-
9	10% Mannitol	2849 ± 1341	0.91 ± 0.20	+	1999 ± 1001	0.92 ± 0.19	+
10	10% Glucose	270 ± 6	0.12 ± 0.03	-	259 ± 4	0.09 ± 0.03	-
11	10% Sucrose	263 ± 5	0.09 ± 0.03	-	267 ± 4	0.09 ± 0.02	-

Table S2. DOX-PDIPG-NP particles lyophilized at different freezing temperatures.

DOX-PDIPG- NP		***− 20 °C***			***− 50 °C***		
Entry	*Conditions*	*Size (Z.ave) nm ± SD*	*PDI ± SD*	*Aggr*	*Size (Z.ave) nm ± SD*	*PDI ± SD*	*Aggr*
1	Before freeze-dry	248 ± 1	0.06 ± 0.02	-	243± 3	0.09 ± 0.01	-
2	No cryoprotectant	2608 ± 790	1.00 ± 0.00	+	693 ± 43	0.81 ± 0.19	+
3	1% Mannitol	446 ± 5	0.40 ± 0.01	+	362 ± 19	0.31 ± 0.06	+
4	1% Glucose	511 ± 22	0.46 ± 0.02	+	290 ± 6	0.18 ± 0.03	+
5	1% Sucrose	583 ± 67	0.50 ± 0.12	+	302 ± 2	0.21 ± 0.01	+
6	5% Mannitol	692 ± 186	0.73 ± 0.04	+	379 ± 10	0.35 ± 0.01	+
7	5% Glucose	247 ± 2	0.10 ± 0.03	-	272 ± 3	0.13 ± 0.01	-
8	5% Sucrose	250 ± 2	0.11 ± 0.03	-	270 ± 7	0.12 ± 0.02	-
9	10% Mannitol	373 ± 45	0.30 ± 0.03	+	345 ± 5	0.25 ± 0.02	+
10	10% Glucose	277 ± 3	0.13 ± 0.03	-	268 ± 1	0.13 ± 0.01	-
11	10% Sucrose	280 ± 2	0.13 ± 0.02	-	263 ± 3	0.11 ± 0.03	-

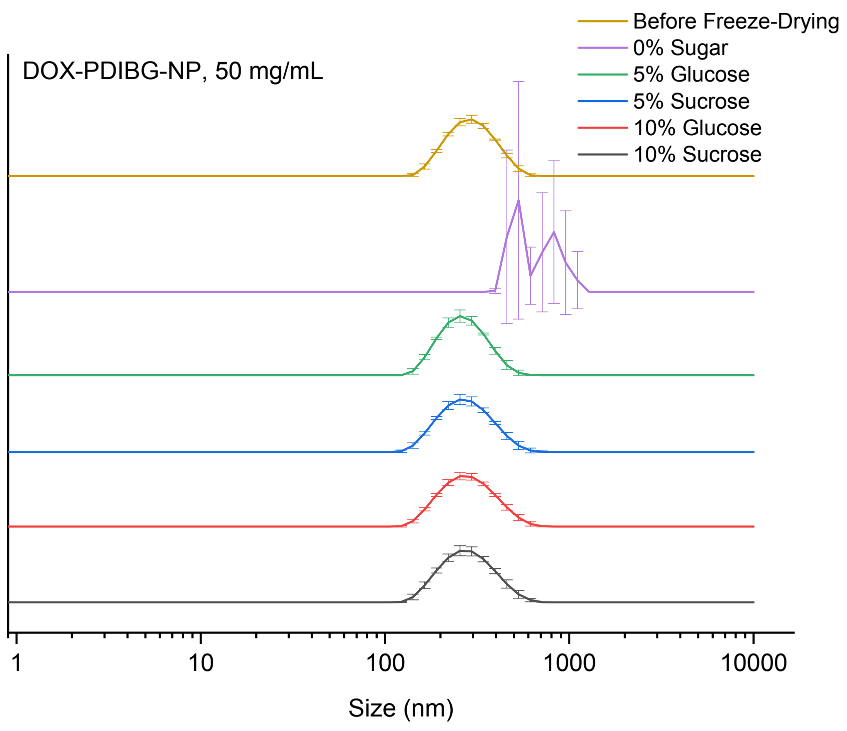

Figure S6. Lyophilized at a concentration of 50 mg/mL, DOX-PDIBG-NPs

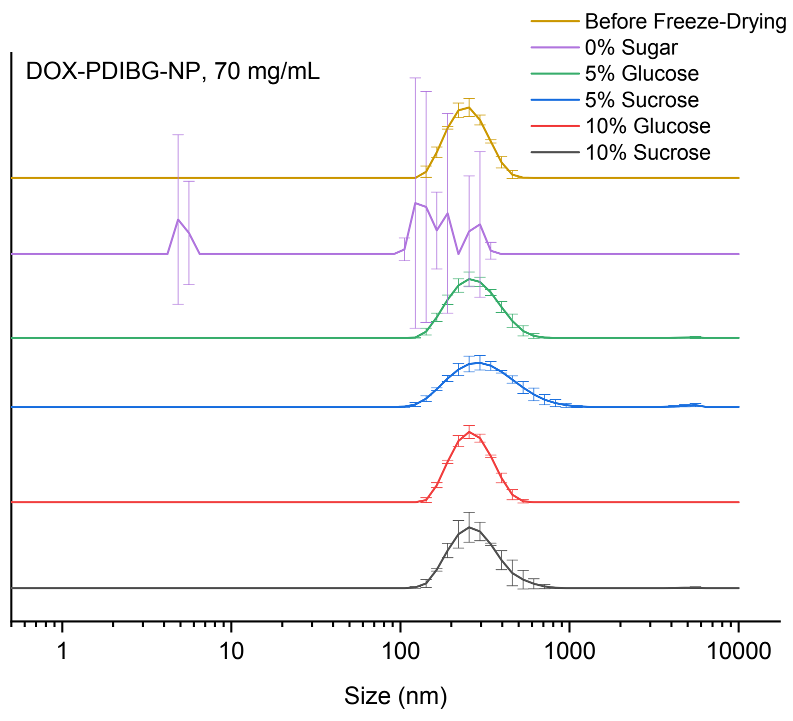

Figure S7. Lyophilized at a concentration of 70 mg/mL, DOX-PDIBG-NPs

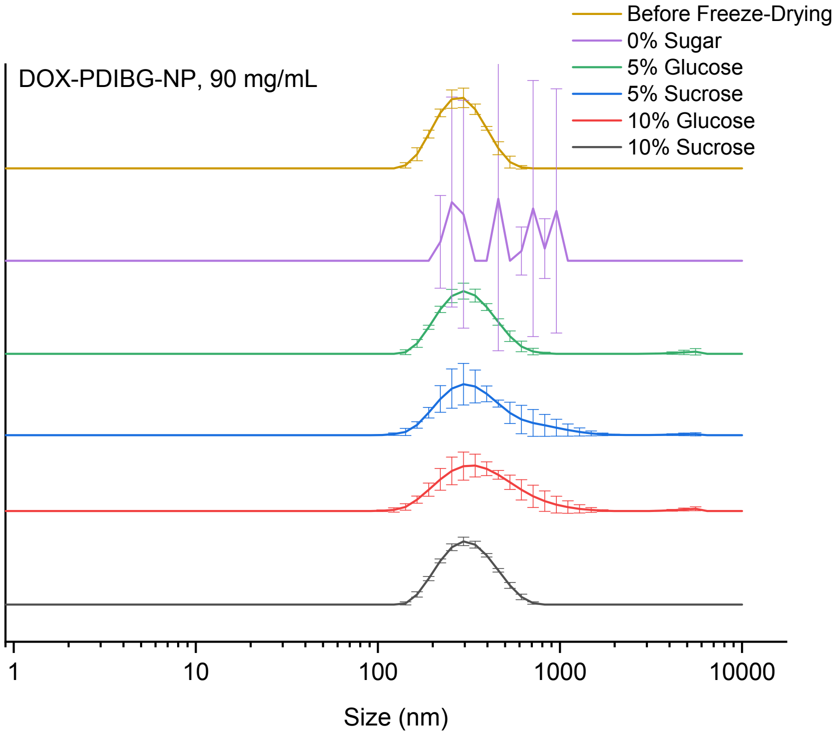

Figure S8. Lyophilized at a concentration of 90 mg/mL, DOX-PDIBG-NPs

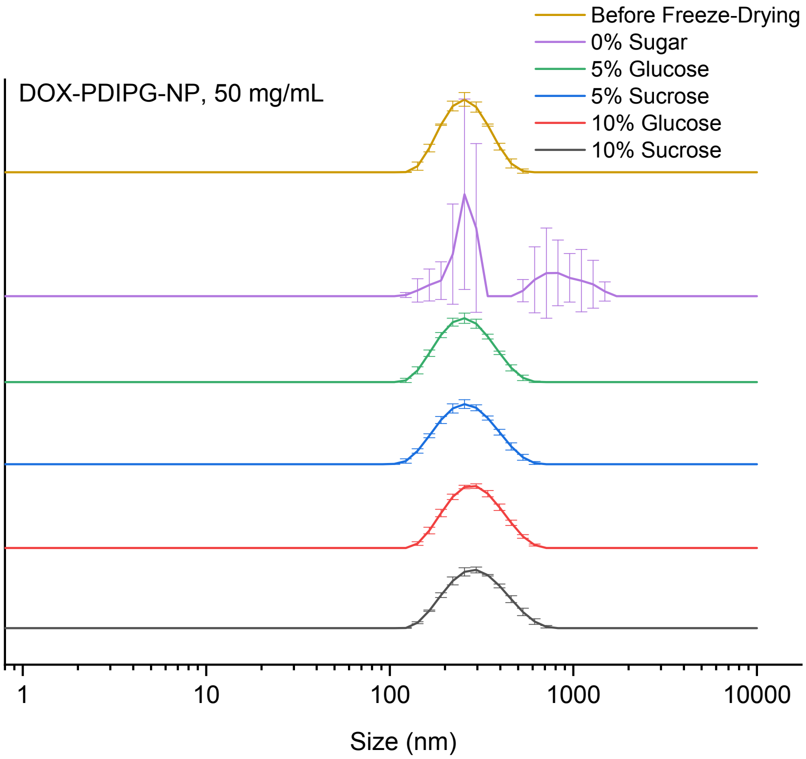

Figure S9. Lyophilized at a concentration of 50 mg/mL, DOX-PDIPG-NPs

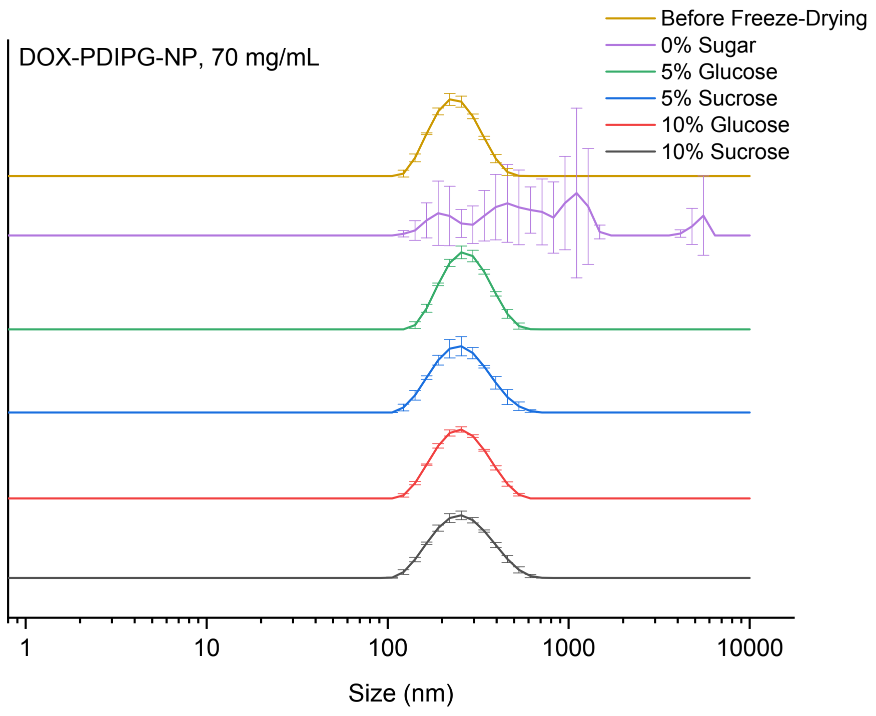

Figure S10. Lyophilized at a concentration of 70 mg/mL, DOX-PDIPG-NPs

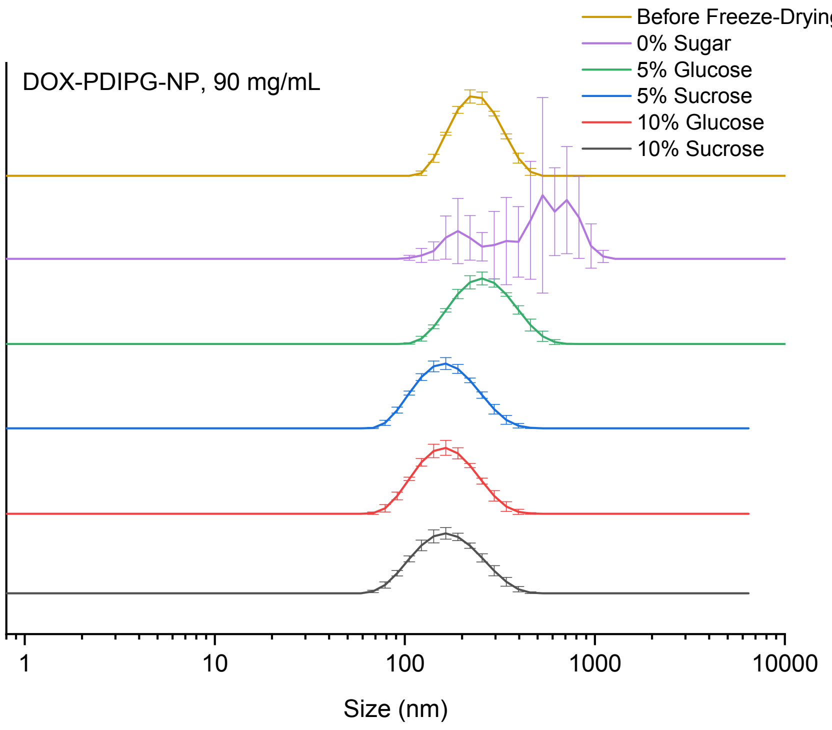

Figure S11. Lyophilized at a concentration of 90 mg/mL, DOX-PDIPG-NPs

Table S3. DOX-PDIBG-NPs lyophilization using different NP:cryo concentrations.

DOX-PDIBG-NP		*50 mg/mL NP Conc.*			*70 mg/mL NP Conc.*			*90 mg/mL NP Conc.*		
Entry	*Conditions*	*Size (Z.ave) nm ± SD*	*PDI ± SD*	*Aggr*	*Size (Z.ave) nm ± SD*	*PDI ± SD*	*Aggr*	*Size (Z.ave) nm ± SD*	*PDI ± SD*	*Aggr*
1	Before freeze-dry	279 ± 2	0.10 ± 0.01	-	240 ± 3	0.05 ± 0.04	-	271 ± 3	0.06 ± 0.02	-
2	No cryoprotectant	2721 ± 1739	0.72 ± 0.31	+	6975 ± 2900	0.95 ± 0.11	+	5165 ± 3164	0.70 ± 0.41	+
3	5% Glucose	254 ± 3	0.08 ± 0.03	-	262 ± 9	0.12 ±0.03	+	307± 8	0.17 ± 0.05	+
4	5% Sucrose	258± 3	0.09 ± 0.02	-	297 ± 7	0.18 ± 0.01	+	334 ± 49	0.17 ± 0.07	+
5	10% Glucose	270 ± 6	0.12 ± 0.03	-	255 ± 2	0.05 ± 0.02	-	343 ± 43	0.23 ± 0.04	+
6	10% Sucrose	263 ± 5	0.09 ± 0.03	-	252 ± 3	0.06 ± 0.02	+	295 ± 5	0.10 ± 0.02	-

Table S4. DOX-PDIPG-NPs lyophilization using different NP:cryo concentrations.

DOX-PDIPG-NP		*50 mg/mL NP Conc.*			*70 mg/mL NP Conc.*			*90 mg/mL NP Conc.*		
Entry	*Conditions*	*Size (Z.ave) nm ± SD*	*PDI ± SD*	*Aggr*	*Size (Z.ave) nm ± SD*	*PDI ± SD*	*Aggr*	*Size (Z.ave) nm ± SD*	*PDI ± SD*	*Aggr*
1	Before freeze-dry	248 ± 1	0.06 ± 0.02	-	227 ± 1	0.07 ± 0.02	-	228 ± 1	0.07 ± 0.01	-
2	No cryoprotectant	2608 ± 790	1 ± 0	+	1412 ± 484	0.76 ± 0.41	+	1515 ± 432	0.86 ± 0.13	+
3	5% Glucose	247 ± 2	0.10 ± 0.03	-	260 ± 3	0.08 ± 0.04	-	243 ± 3	0.10 ± 0.02	-
4	5% Sucrose	250 ± 2	0.11 ± 0.03	-	240 ± 2	0.09 ± 0.03	-	247 ± 2	0.13 ± 0.03	-
5	10% Glucose	277 ± 3	0.13 ± 0.03	-	242 ± 1	0.09 ± 0.01	-	243 ± 1	0.10 ± 0.03	-
6	10% Sucrose	280 ± 2	0.13 ± 0.02	-	243 ± 3	0.10 ± 0.01	-	245± 2	0.12 ± 0.02	-

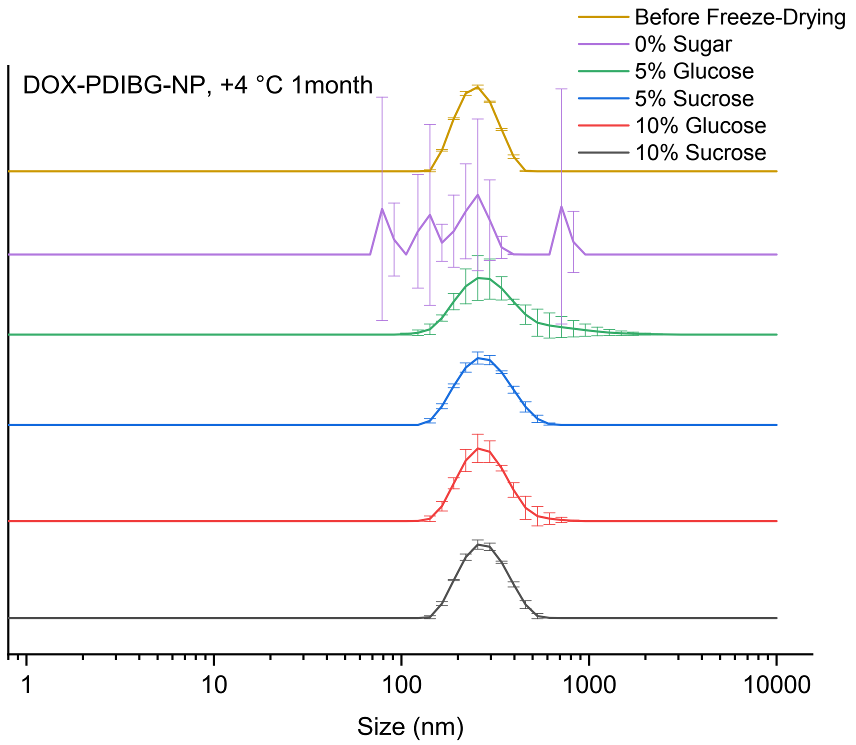

Figure S12. Storage of lyophilized DOX-PDIBG-NPs at +4 °C for 1 month

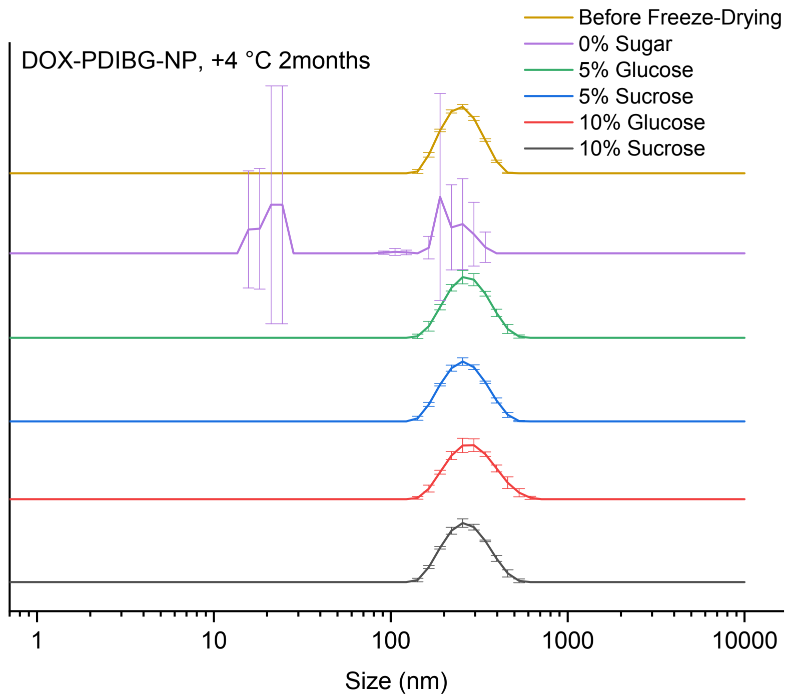

Figure S13. Storage of lyophilized DOX-PDIBG-NPs at +4 °C for 2 months

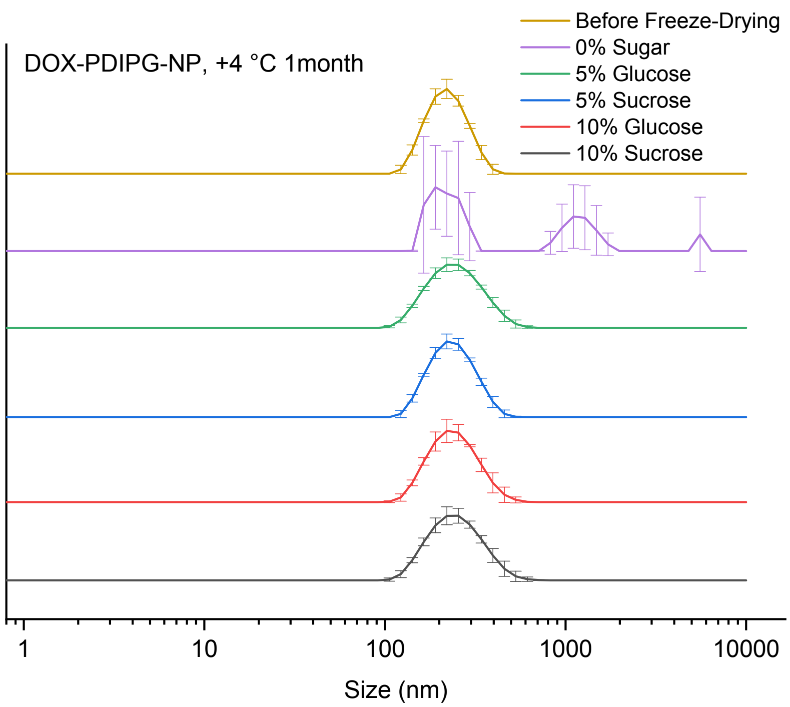

Figure S14. Storage of lyophilized DOX-PDIPG-NPs at +4 °C for 1 month

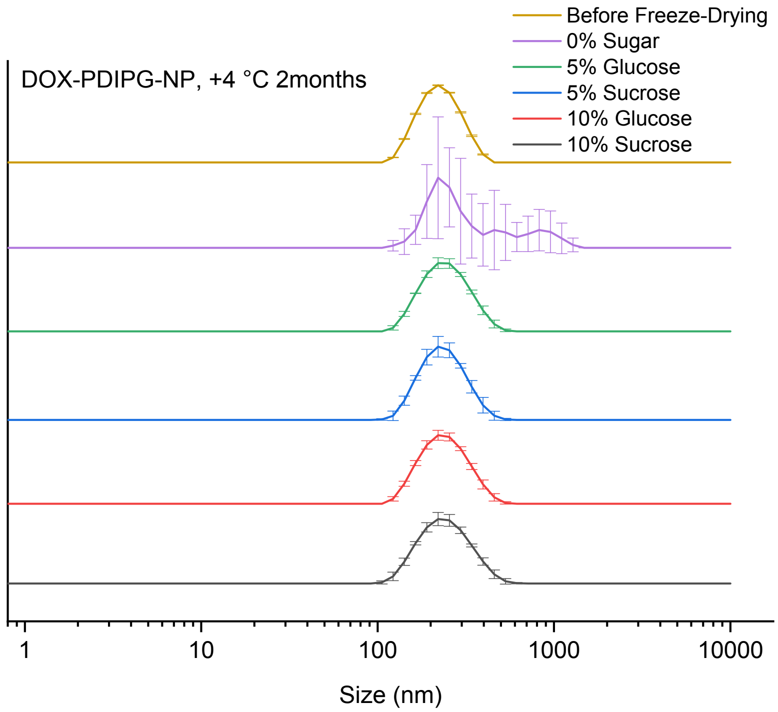

Figure S15. Storage of lyophilized DOX-PDIPG-NPs at +4 °C for 2 months

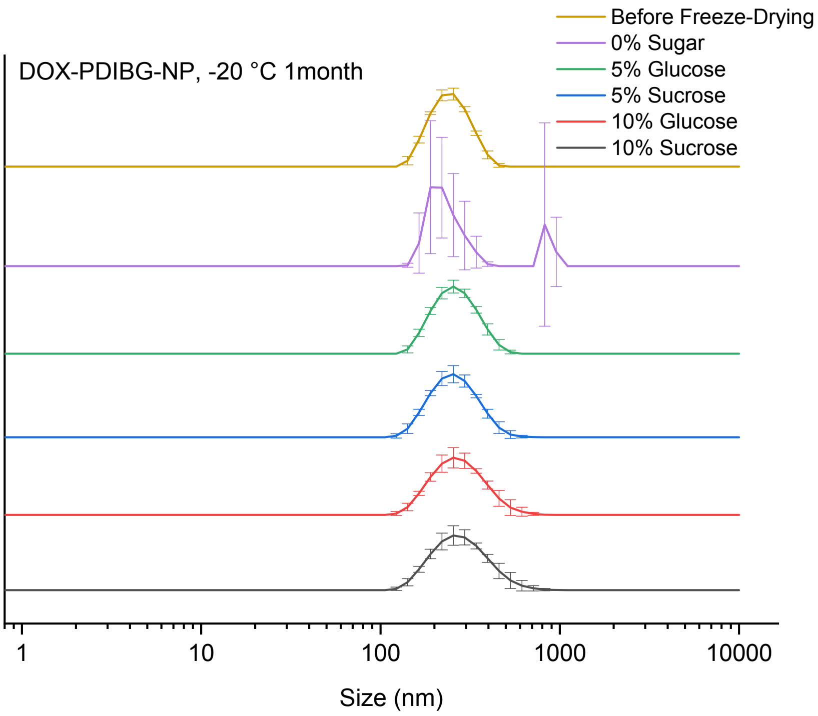

Figure S16. Storage of lyophilized DOX-PDIBG-NPs at -20 °C for 1 month

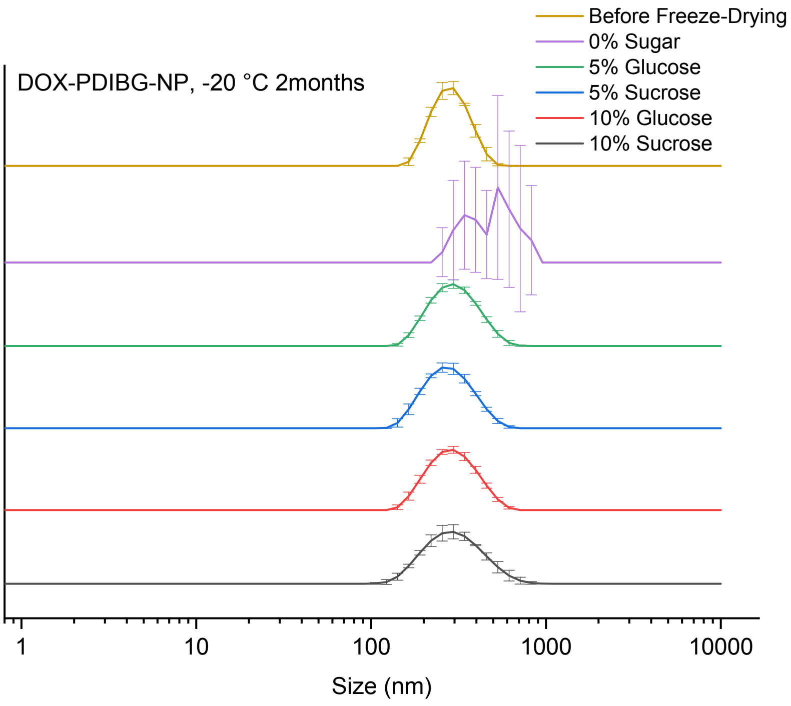

Figure S17. Storage of lyophilized DOX-PDIBG-NPs at -20 °C for 2 months

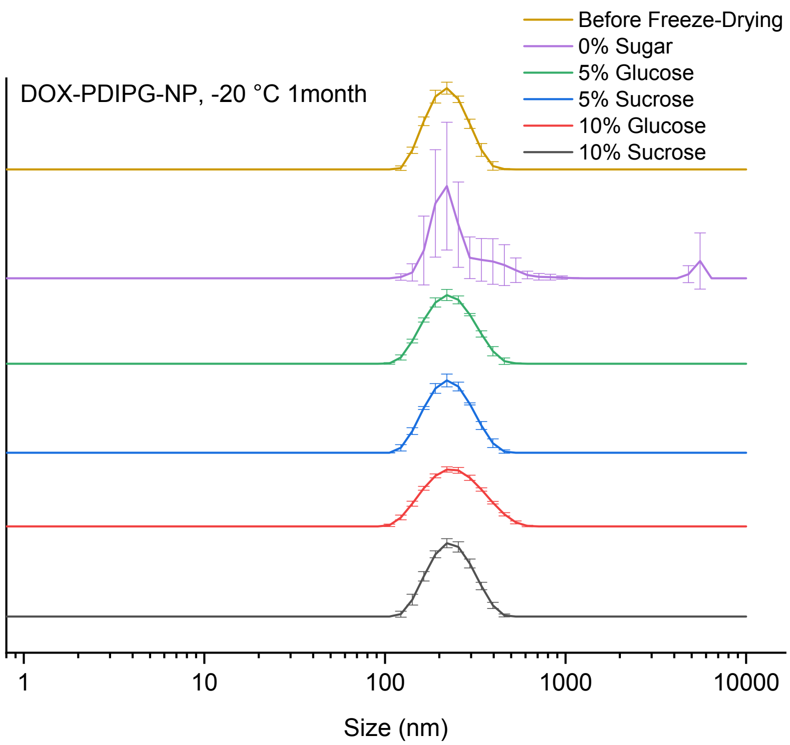

Figure S18. Storage of lyophilized DOX-PDIPG-NPs at -20 °C for 1 month

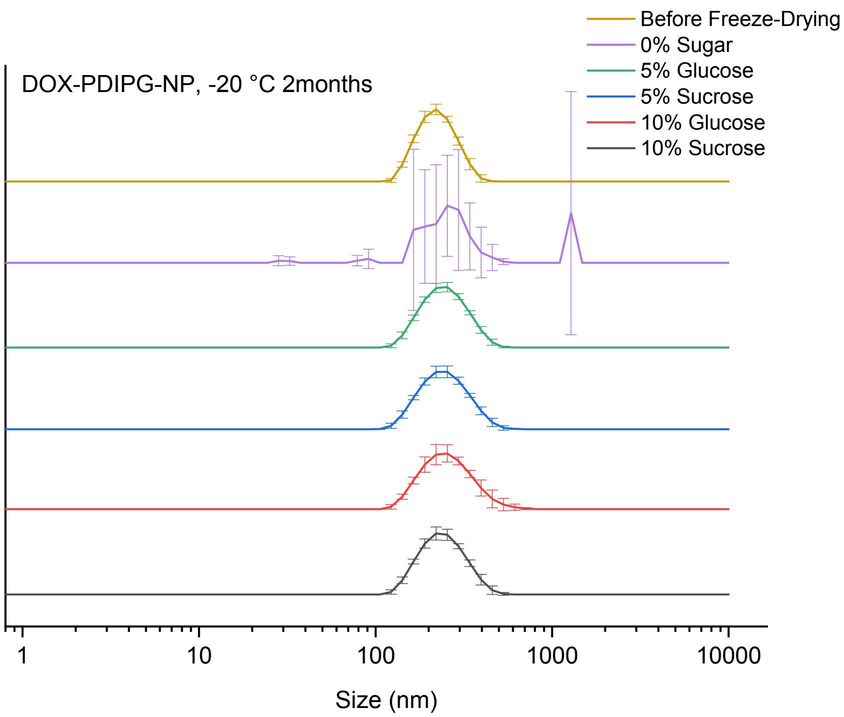

Figure S19. Storage of lyophilized DOX-PDIPG-NPs at -20 °C for 2 months

Table S5. Storage of lyophilized DOX-PSG-NPs at 4 °C for 2 months.

Entry	Type of PSG	*Conditions*	***4 °C, 1 month***			***4 °C, 2 months***		
			Size (Z.ave) nm ± SD	*PDI ± SD*	*Aggr*	*Size (Z.ave) nm ± SD*	*PDI ± SD*	*Aggr*
1	PDIBG	Before freeze-dry	245 ± 1	0.03 ± 0.01	-	243 ± 3	0.03 ± 0.01	-
2	PDIPG		249 ± 1	0.06 ± 0.03	-	216 ± 1	0.05 ± 0.01	-
3	PDIBG	No cryoprotectant	2547 ± 2908	0.69 ± 0.31	+	4654 ± 2910	0.94 ± 0.10	+
4	PDIPG		2360 ± 1903	0.89 ± 0.27	+	1138 ± 416	0.90 ± 0.13	+
5	PDIBG	5% Glucose	287 ± 34	0.13 ± 0.08	-	261± 4	0.06 ± 0.03	-
6	PDIPG		230 ± 4	0.09 ± 0.02	-	231 ± 1	0.09 ± 0.02	-
7	PDIBG	5% Sucrose	263± 4	0.09 ± 0.02	-	251 ± 2	0.06 ± 0.01	-
8	PDIPG		226± 2	0.06 ± 0.02	-	224 ± 4	0.07 ± 0.02	-
9	PDIBG	10% Glucose	266 ± 18	0.07 ± 0.05	-	274 ± 5	0.09 0.04	-
10	PDIPG		229 ± 7	0.08 ± 0.03	-	227 ± 4	0.08 0.01	-
11	PDIBG	10% Sucrose	263 ± 2	0.07 ± 0.01	-	257 ± 2	0.06 ± 0.02	-
12	PDIPG		235± 6	0.10 ± 0.04	-	227 ± 1	0.11 ± 0.02	-

Table S6. Storage of lyophilized DOX-PSG-NPs at -20 °C for 2 months.

Entry	Type of PSG	*Conditions*	***–20 °C, 1 month***			***–20 °C, 2 months***		
			Size (Z.ave) nm ± SD	*PDI ± SD*	*Aggr*	*Size (Z.ave) nm ± SD*	*PDI ± SD*	*Aggr*
1	PDIBG	Before freeze-dry	235 ± 2	0.06 ± 0.03	-	249 ± 2	0.02 ± 0.02	-
2	PDIPG		215 ± 4	0.04 ± 0.02	-	218 ± 1	0.05 ± 0.01	-
3	PDIBG	No cryoprotectant	2882 ± 1320	0.94 ± 0.11	+	3107± 1061	0.85 ± 0.17	+
4	PDIPG		2121 ± 1976	0.88 ± 0.20	+	5207 ± 3119	0.76 ± 0.27	+
5	PDIBG	5% Glucose	250 ± 5	0.07 ± 0.02	-	264 ± 6	0.08 ± 0.02	-
6	PDIPG		218 ± 2	0.07 ± 0.02	-	234± 4	0.07 ± 0.01	-
7	PDIBG	5% Sucrose	245 ± 2	0.07 ± 0.03	-	268 ± 4	0.11 ± 0.04	-
8	PDIPG		218 ± 2	0.05 ± 0.02	-	232 ± 3	0.09 ± 0.03	-
9	PDIBG	10% Glucose	261 ± 12	0.11 ± 0.05	-	287 ± 17	0.12 ± 0.04	-
10	PDIPG		229 ± 2	0.12 ± 0.02	-	241 ± 12	0.09 ± 0.04	-
11	PDIBG	10% Sucrose	262 ± 13	0.11 ± 0.04	-	270± 2	0.10 ± 0.02	-
12	PDIPG		223 ± 6	0.06 ± 0.02	-	229 ± 4	0.08 ± 0.01	-

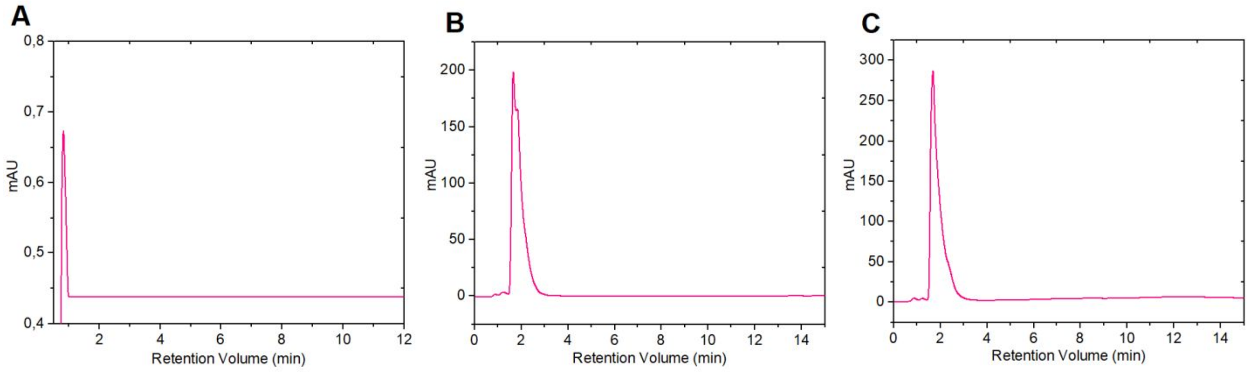

Figure S20. HPLC spectrum of MeOH (A), LDIBG (B) and LDIPG (C) monomers (4 mg/mL) in MeOH at 200 nm
